# Confirmation of COVID-19 infection status and reporting of Long COVID symptoms in a population-based birth cohort: No evidence of a nocebo effect

**DOI:** 10.1177/13591053241228711

**Published:** 2024-01-25

**Authors:** Catherine IA Macleod-Hall, Marcus R Munafò, Maddy L Dyer

**Affiliations:** 1 University Hospitals Bristol and Weston NHS Foundation Trust, UK; 2 University of Bristol, UK; 3 Medical Research Council Integrative Epidemiology Unit at the University of Bristol, UK; 4 National Institute for Health Research Bristol Biomedical Research Centre, UK

**Keywords:** ALSPAC, children of the 90s, coronavirus, Long COVID, nocebo, pandemic

## Abstract

Some patients with COVID-19 develop symptoms after the acute infection, known as ‘Long COVID’. We examined whether or not confirmation of COVID-19 infection status could act as a nocebo, using data from questionnaires distributed to the Avon Longitudinal Study of Parents and Children cohort. We examined associations between confirmation of COVID-19 infection status (confirmed by a positive test vs unconfirmed) and reporting of Long COVID symptoms. We explored the roles of sex and anxiety as potential moderators. There was no clear evidence of a strong association between confirmation of COVID-19 infection status and the Long COVID composite score, physical or psychological symptoms or duration of symptoms. There was no clear evidence of moderation by sex or anxiety. We therefore found no evidence of a nocebo effect. Our data suggest that this psychological mechanism does not play a role in the medical symptomatology experienced by patients with Long COVID.

## Background

The coronavirus disease (COVID-19) is an infectious disease caused by the severe acute respiratory syndrome coronavirus-2 (SARS-CoV-2) (World Health Organization, 2021). Whilst most people recover within 2 weeks (Raveendran et al., 2021), many continue to have symptoms related to COVID-19 after the acute phase of illness, referred to as ‘Long COVID’ (Yong, 2021). The National Institute for Health and Care Excellence (NICE) defines Long COVID as signs and symptoms that continue or develop after ‘acute COVID-19’ (0–4 weeks). It includes both ‘ongoing symptomatic COVID-19’ (4–12 weeks) and ‘post-COVID-19 syndrome’ (>12 weeks) (NICE, 2020). The Office for National Statistics (ONS) estimates that 1.9 million people living in the UK were experiencing self-reported Long COVID in March 2023 (ONS, 2023). Fatigue, dyspnoea and myalgia are the most common symptoms (ONS, 2023).

The aetiology underlying Long COVID is currently unknown. Biological theories have been proposed. For example, Long COVID may be driven by the viral reservoirs, long-term organ damage and/or immune dysregulation (Marx, 2021; Nalbandian et al., 2021; Xie et al., 2022). There is conflicting evidence as to whether the likelihood of developing Long Covid is associated with greater severity of the acute COVID-19 illness (Sudre et al., 2021; Whitaker et al., 2021) or not (Sykes et al., 2021; Townsend et al., 2020). This suggests that there may be contributory aetiological mechanisms that are independent to some degree from SARS-CoV-2. Long COVID is associated with female sex (Davis et al., 2023; Sykes et al., 2021; Thompson et al., 2022; Townsend et al., 2020), pre-pandemic anxiety, depression and poorer general health (Thompson et al., 2022; Townsend et al., 2020), older age (BMJ, 2020; Thompson et al., 2022), higher body mass index (BMI) (Sudre et al., 2021; Sykes et al., 2021; Whitaker et al., 2021) and white ethnicity (Thompson et al., 2022). However, it is currently unclear whether any of these factors are causal, and if so, what the mechanisms are that they act through. Long COVID has been likened to myalgic encephalomyelitis/chronic fatigue syndrome (ME/CFS) (Hunt et al., 2022; Poenaru et al., 2021; Wong and Weitzer, 2021), which exists under the umbrella of medically unexplained symptoms despite the cumulating evidence for biological mechanisms (Cortes Rivera et al., 2019). Similarities extend to the symptoms experienced (Davis et al., 2021; Twomey et al., 2022) and possible shared underlying mechanisms (Davis et al., 2023).

Psychosocial mechanisms may also contribute to the aetiology of Long COVID. Sykes et al. (2021) argue that the biopsychosocial effects of COVID-19 may lead to physical and psychological symptoms akin to post-traumatic syndromes. However, some are concerned about proposed psychological mechanisms of Long COVID, arguing that they could perpetuate the idea of medical symptomatology in the absence of biological cause, as with the pre-existing debate about ME/CFS (Hunt et al., 2022). For example, some patients report that their concerns have been dismissed as symptoms related to anxiety and stress (medical gaslighting) (Yong, 2021). In the case of ME/CFS, discourse focused on psychological mechanisms can be a source of conflict between patients and their physicians, and can lead to harmful interventions (Geraghty and Blease, 2019). It is therefore important to determine the presence or absence of nocebo effects in Long COVID, to inform these discussions and provide guidance for clinicians.

The nocebo effect is ‘the induction or worsening of symptoms induced by sham or active therapies’ (Planès et al., 2016). Psychological mechanisms (e.g. negative expectations) and neurobiological mechanisms (e.g. the hypothalamus–pituitary–adrenal axis) can produce adverse events (Planès et al., 2016). Negative expectations of long-term symptoms following an acute COVID-19 infection could increase anticipatory anxiety and, in turn, contribute to the symptoms experienced (Karnatovskaia et al., 2020; Planès et al., 2016). Amanzio et al. (2020) suggest that ‘the COVID-19 era is an unavoidable breeding ground for the possible nocebo effect’. Like Long COVID, nocebo effects appear to be more common in women (Casper et al., 2001; Klosterhalfen et al., 2009) and in people with anxiety and depression (Amanzio et al., 2020; Wells and Kaptchuk, 2012). One study found that certainty of being infected with COVID-19 and anxiety were associated with perceived severity of acute COVID-19 symptoms, which the authors attributed to a nocebo effect (Daniali and Flaten, 2022). Additionally, another study found that baseline beliefs regarding COVID-19 symptom severity predicted subsequent reporting of COVID-19-like symptoms in individuals without a positive test (Rozenkrantz et al., 2022). Similarly, confirmation of being infected with COVID-19 may act as a nocebo and people may attribute psychological and somatic sensations as Long COVID symptoms.

This study explored the possible influence of nocebo effects on Long COVID via the following aims: to what degree (1) confirmation of COVID-19 infection status (confirmed vs unconfirmed) is associated with Long COVID symptoms (i.e. duration of COVID-19 symptoms, physical symptoms and psychological symptoms), (2) sex and anxiety are possible moderators of any association between confirmation of COVID-19 infection status and reporting of Long COVID symptoms and (3) COVID-19 infection status is associated with reporting of Long COVID symptoms. We hypothesised that among participants with confirmed (vs unconfirmed) COVID-19 infection status: (1) number of Long COVID symptoms (a composite score of physical and psychological symptoms) would be higher, (2) number of physical Long COVID symptoms would be higher, (3) anxiety and depression would be higher, and wellbeing would be lower and (4) presence of ongoing COVID-19 (symptoms >4 weeks) and post-COVID-19 syndrome (symptoms >12 weeks) would be greater. We also hypothesised that (5) the magnitude of any associations would be greater among participants who are female and participants who reported having anxiety at the start of the pandemic and (6) number of Long COVID symptoms would be higher among participants who reported having had COVID-19 compared to participants who reported not having had COVID-19.

## Methods

### Design

We conducted analyses of secondary data from the Avon Longitudinal Study of Parents and Children (ALSPAC), a prospective, population-based birth cohort study (Boyd et al., 2013; Fraser et al., 2013; Northstone et al., 2019, 2023). We used data from the four online COVID-19 questionnaires delivered to the ALSPAC cohort from April to May 2020, May to July 2020, October 2020 and November 2020 to March 2021 (Northstone et al., 2020a, 2020b). Study data were collected and managed using REDCap electronic data capture tools hosted at the University of Bristol (Harris et al., 2009). The study website contains details of all the data that are available (http://www.bristol.ac.uk/alspac/researchers/our-data/). Ethics approval was obtained from the ALSPAC Ethics and Law Committee and the Local Research Ethics Committees (http://www.bristol.ac.uk/alspac/researchers/research-ethics/). Written informed consent for the use of data collected via questionnaires was obtained from participants following the recommendations of the ALSPAC Ethics and Law Committee at the time. We pre-registered the study protocol on the Open Science Framework (https://osf.io/h5juf).

### Participants

Pregnant women resident in Avon, UK with expected dates of delivery from 1st April 1991 to 31st December 1992 were invited to take part in the ALSPAC study. When the oldest children were approximately 7 years old, an attempt was made to bolster the initial sample. As a result, the total sample size for analyses using any data collected after the age of seven is therefore 14,901 children that were alive at 1 year of age. Questionnaires 1, 2 and 4 were distributed by email to all ALSPAC participants with an active email address (Northstone et al., 2020a, 2020b; Smith et al., 2021). Questionnaire 3 was distributed with home-based antibody testing, and so an initial invitation was distributed by email to respondents to questionnaire 1, and respondents to questionnaire 2 that indicated in interest in taking part in biological sampling (Northstone et al., 2021). We used data from ALSPAC young adults (G1), mothers (G0) and mother’s partners (G0) to maximise sample size. ALSPAC participants were offered entry into a prize draw (three prizes of £100) for each questionnaire they returned. G1 participants were also offered a £10 shopping voucher for completion of questionnaire 4, as it was embedded within their annual questionnaire for which they usually receive an incentive.

### Measures

The measures used are described below and in Table S1.

#### Exposure variables

At each questionnaire, participants self-reported having had COVID-19 with four response options: ‘yes, confirmed by a positive test’, ‘yes, suspected by a doctor but not tested’, ‘yes, my own suspicions’ or ‘no’. The primary exposure variable was ‘confirmation of COVID-19 infection status’, for which ‘yes, confirmed by a positive test’ responses were classified as ‘confirmed COVID-19’, and ‘yes, suspected by a doctor but not tested’ or ‘yes, my own suspicions’ responses were classified as ‘unconfirmed COVID-19’. The secondary exposure variable was ‘COVID-19 infection status’ (no vs yes). ‘Yes, confirmed by a positive test’, ‘yes, suspected by a doctor but not tested’ and ‘yes, my own suspicions’ were classified as ‘yes’, and ‘no’ responses as ‘no’.

#### Outcome variables

A continuous ‘Long COVID composite’ variable was created by summing the number of physical and psychological symptoms. Presence of each symptom was scored as 1. For the psychological symptoms this included likely depression (Short Mood and Feelings Questionnaire (SMFQ) ⩾12 (Messer et al., 1995)), moderate/severe anxiety (Generalised Anxiety Disorder-7 questionnaire (GAD-7) ⩾10 (Spitzer et al., 2006)) and low wellbeing (Warwick-Edinburgh Mental Well-being Scale (WEMWS) ⩽40 (Tennant et al., 2007)). The constituent physical and psychological symptoms variables are described further below.

Participants were asked to report the presence of physical symptoms (Table S1) each month between October 2019 and March 2021 in questionnaires 1, 3 and 4. The total number of symptoms a participant reported within the relevant timeframe were summed to create a continuous variable. As a deviation from the protocol, we excluded symptoms that did not have data available for the entire study period. For participants who reported having had COVID-19, we summed physical symptoms in the ongoing COVID-19 (‘physical symptoms at 4–12 weeks’) and post-COVID-19 syndrome (‘physical symptoms at 12–20 weeks’) timeframes, in relation to their reported date of COVID-19 illness. A timeframe was selected for participants without COVID-19 infection (Table S1).

Questionnaires 1, 2 and 4 assessed ‘depression’, ‘anxiety’ and ‘wellbeing’ within the 2 weeks before questionnaire completion, using the SMFQ (Messer et al., 1995), GAD-7 (Spitzer et al., 2006) and the WEMWS (Tennant et al., 2007) questionnaires, respectively. These were used as continuous outcome variables.

Finally, two ‘duration of symptoms’ variables were created. In questionnaire 4, participants reported the duration of symptoms they attributed to COVID-19. For the variable ‘ongoing COVID-19’, participants who reported symptoms lasting 4 or more weeks were classified as having had ongoing COVID-19, and those who reported COVID-19 symptoms lasting 1 day to 4 weeks were classified as having had acute COVID-19. For the variable ‘post-COVID-19’, participants who reported symptoms lasting 12 or more weeks were classified as having had post-COVID-19 syndrome, and those who reported COVID-19 symptoms lasting 1 day to 12 weeks were classified as having had acute or ongoing COVID-19.

#### Moderator variables

Sex (male vs female) and anxiety (no vs yes) were selected as moderator variables. Presence of anxiety reported during questionnaire 1 assessed anxiety status.

#### Covariates

Analyses were adjusted for age, sex, BMI, white ethnic group and anxiety and comorbidities at the start of the pandemic. Covariates were selected based on their a priori relevance and/or their associations with Long COVID in the literature (i.e. their potential to be a confounder).

### Statistical analyses

Data were analysed using Stata MP (version 16). Analyses of the whole sample (G0 and G1 cohorts combined) accounted for relatedness within the model (i.e. standard errors allowed for intragroup correlation, relaxing the independence of observations assumption). Analyses were performed on available data. Associations between confirmation of COVID-19 infection status (unconfirmed vs confirmed) and the long COVID outcomes were examined using linear regression: the Long COVID composite; physical symptoms at 4–12 weeks; physical symptoms at 12–20 weeks; depression symptoms in the last 2 weeks; anxiety symptoms in the last 2 weeks and wellbeing in the last 2 weeks (aim 1, hypotheses 1, 2 and 3). Associations between confirmation of COVID-19 infection status and the reported presence of ongoing COVID-19 and post-COVID-19 syndrome (aim 1, hypothesis 4) were examined using logistic regression.

Moderation by sex and anxiety (i.e. interaction between confirmation of COVID-19 infection status and sex/anxiety; aim 2, hypotheses 4 and 5) was examined in several stages. First, unadjusted analyses were stratified by sex (male vs female) and anxiety (no vs yes). Using linear regression for continuous outcomes and logistic regression for dichotomous outcomes, three models assessed the interaction between confirmation of COVID-19 infection status and moderator variables on each Long COVID outcome. Model A examined the unadjusted interaction (i.e. including a confirmation of COVID-19 infection status × sex interaction term and a confirmation of COVID-19 infection status × anxiety interaction term). Model B examined the association between confirmation of having had COVID-19 and Long COVID outcomes, adjusted for moderator variables (i.e. sex or anxiety). Model C examined the interaction between confirmation of having had COVID-19 and moderator variables on Long COVID outcomes, adjusted for covariates. Models A and B were compared using likelihood ratio tests.

Associations between COVID-19 infection status and the Long COVID outcomes described above were explored using linear regression (aim 3, hypothesis 6). Interaction tests (as described above for hypothesis 5) were also performed for the exposure COVID-19 infection status and Long COVID outcomes.

For all analyses, impact of potential confounding variables was assessed by comparing unadjusted and adjusted models. Adjusted results are reported in the text unless stated otherwise. For moderation analyses, Model C results are reported. Complete case analyses (sensitivity analyses) were performed to tease apart possible effects of confounding vs reductions in sample size between unadjusted and adjusted models. Strength of evidence against the null hypothesis was interpreted in the following way: *p* < 0.05 provides modest evidence and *p* < 0.001 provides strong evidence (Sterne and Davey-Smith, 2001). The minimal clinically important difference was considered to be an unstandardised coefficient (B) of more than ±2 for all continuous outcomes and an odds ratio (OR) of ⩽0.5 or ⩾1.5 for the duration of symptoms variables.

## Results

### Participants

In total, 6807 participants responded to questionnaire 1 (Northstone et al., 2020a), 6482 to questionnaire 2 (Northstone et al., 2020b), 4819 to questionnaire 3 (Northstone et al., 2021) and 8643 to questionnaire 4 (Smith et al., 2021; Table S2). In total, 330 participants reported confirmed COVID-19, 1700 reported unconfirmed COVID-19 and 7534 reported not having had COVID-19 to their knowledge. Sample sizes ranged from 341 to 638 for analyses using the confirmation of COVID-19 infection status exposure variable (aims 1 and 2), and from 618 to 8134 for analyses using the COVID-19 infection status exposure variable (aim 3). Of participants that had data for the main exposure variable, confirmation of COVID-19 infection status (*n* = 2030), ages ranged from 31 to 81 (mean 59, standard deviation (SD) 4.9), 71% were female, and 97% were White. Six hundred sixty-four were G0 mothers, 204 were G0 fathers, 1157 were G1 offspring and 25 were G1 partners.

### Associations between confirmation of COVID-19 infection status and Long COVID outcomes (aim 1)

Descriptive statistics for outcomes in each level of the exposure variable are reported in Table 1 with tests of difference between groups (*t*-tests for continuous outcomes and chi-squared tests for dichotomous outcomes). Regression results for the Long COVID composite and physical and psychological symptoms are reported in Table 2. There was no clear evidence of an association between confirmation of COVID-19 infection status and the Long COVID composite (*B* 0.10, 95% confidence interval (CI) −2.26 to 2.46, *p* = 0.935), however the width of the confidence interval is compatible with a clinically significant result in either direction. There was no clear evidence of an association between confirmation of COVID-19 infection status and physical symptoms (4–12 weeks: *B* −0.65, 95% CI −1.49 to 0.19, *p* = 0.128; 12–20 weeks: *B* −0.01, 95% CI −1.02 to 1.01, *p* = 0.998), depression (*B* −0.30, 95% CI −1.39 to 0.78, *p* = 0.583) or anxiety (*B* −0.33, 95% CI −1.28 to 0.62, *p* = 0.500). There was no clear evidence of an association between confirmation of COVID-19 infection status and wellbeing (*B* 1.43, 95% CI −0.59 to 3.44, *p* = 0.165), however the confidence interval indicates a possible clinically significant increase in wellbeing. Finally, there was no clear evidence of an association between confirmation of COVID-19 infection status and duration of symptoms (ongoing COVID-19: OR 0.78, 95% CI 0.58–1.06, *p* = 0.116; post-COVID-19 syndrome: OR 0.68, 95% CI 0.43–1.05, *p* = 0.085) (Table 3).

**Table 1.** Confirmation of COVID-19 infection status and Long COVID outcomes.

		*n*	Unconfirmed COVID-19	Confirmed COVID-19	*p*-Value
Long COVID composite, mean (SD)		451	7.1 (9.8)	6.6 (9.6)	0.636
Duration of symptoms, *N* (%)					
Ongoing COVID-19	No	1146	606 (72)	240 (77)	0.116
	Yes		229 (27)	71 (23)	
Post-COVID-19 syndrome	No	1146	732 (88)	284 (91)	0.083
	Yes		103 (12)	27 (8.7)	
Physical symptoms, mean (SD)					
Physical symptoms at 4–12 weeks		756	3.2 (5.0)	2.4 (4.6)	0.025
Physical symptoms at 12–20 weeks		543	2.8 (4.6)	2.4 (4.4)	0.376
Psychological symptoms, mean (SD)					
Depression		527	5.2 (5.1)	5.9 (6.4)	0.170
Anxiety		698	5.4 (4.9)	5.5 (5.3)	0.743
Wellbeing		694	45.4 (8.9)	45.9 (9.6)	0.577

Confirmed COVID-19, coded ‘1’, is confirmed by any test; unconfirmed COVID-19, coded ‘0’, is suspected by participant or doctor but not tested. Long COVID composite is a composite of physical symptoms reported at 4–20 weeks after onset of COVID-19 infection, and psychological symptoms. Ongoing COVID-19: symptoms 4 weeks or more after the onset of the original COVID-19 infection. Post-COVID-19 syndrome: symptoms 12 weeks or more after the onset of the original COVID-19 infection. Depression symptoms measured using the Short Mood and Feelings Questionnaire (SMFQ), anxiety symptoms measured using the Generalised Anxiety Disorder-7 questionnaire (GAD-7) and wellbeing measured using the Warwick-Edinburgh Mental Well-being Scale (WEMWS). *T*-tests were used to assess differences between groups for continuous outcomes, and chi-squared tests for dichotomous outcomes.

SD: standard deviation.

**Table 2.** Associations between confirmation of COVID-19 infection status and reported physical and psychological symptoms.

	Unadjusted			Adjusted		
	*n*	*B* (95% CI)	*p*-Value	*n*	*B* (95% CI)	*p*-Value
Long COVID composite	451	−0.50 (−2.53 to 1.54)	0.632	341	0.10 (−2.26 to 2.46)*	0.935
Physical symptoms						
Physical symptoms at 4–12 weeks	756	−0.82 (−1.53 to −0.12)	0.022	523	−0.65 (−1.49 to 0.19)	0.128
Physical symptoms at 12–20 weeks	543	−0.39 (−1.25 to 0.46)	0.366	403	−0.01 (−1.02 to 1.01)	0.998
Psychological symptoms						
Depression	687	0.67 (−0.42 to 1.76)	0.225	477	−0.30 (−1.39 to 0.78)	0.583
Anxiety	698	0.15 (−0.77 to 1.06)	0.752	484	−0.33 (−1.28 to 0.62)	0.500
Wellbeing	694	0.45 (−1.20 to 2.10)	0.591	482	1.43 (−0.59 to 3.44)*	0.165

An asterisk (*) denotes an adjusted model for which the confidence intervals are consistent with a clinically significant result however our study was underpowered to resolve this. Linear regressions. Adjusted analyses are adjusted for age, sex, body mass index, ethnicity, anxiety and comorbidities at the start of the pandemic. Confirmed COVID-19, coded ‘1’, is confirmed by any test; unconfirmed COVID-19, coded ‘0’, is suspected by participant or doctor but not tested. Long COVID composite is a composite of physical symptoms reported at 4–20 weeks after onset of COVID-19 infection, and psychological symptoms. Depression symptoms measured using the Short Mood and Feelings Questionnaire (SMFQ), anxiety symptoms measured using the Generalised Anxiety Disorder-7 questionnaire (GAD-7) and wellbeing measured using the Warwick-Edinburgh Mental Well-being Scale (WEMWS).

95% CI: 95% confidence intervals; *B*: unstandardised coefficients.

**Table 3.** Associations between confirmation of COVID-19 infection status and duration of COVID-19 symptoms.

	Unadjusted			Adjusted		
	*n*	Odds ratio (95% CI)	*p*-Value	*n*	Odds ratio (95% CI)	*p*-Value
Ongoing COVID-19	1146	0.78 (0.58–1.06)	0.116	638	0.90 (0.58–1.38)	0.618
Post-COVID-19 syndrome	1146	0.68 (0.43–1.05)	0.085	638	0.76 (0.40–1.42)	0.388

Logistic regressions. Adjusted analyses are adjusted for age, sex, body mass index, ethnicity, anxiety and comorbidities at the start of the pandemic. Confirmed COVID-19, coded ‘1’, is confirmed by any test; unconfirmed COVID-19, coded ‘0’, is suspected by participant or doctor but not tested. Ongoing COVID-19: symptoms 4 weeks or more after the onset of the original COVID-19 infection. Post-COVID-19 syndrome: symptoms 12 weeks or more after the onset of the original COVID-19 infection.

95% CI: 95% confidence intervals.

### Moderation by sex and anxiety on the relationship between confirmation of COVID-19 infection status and Long COVID outcomes (aim 2)

There was no clear evidence of a stronger association between confirmation of COVID-19 infection status and the Long COVID composite, physical symptoms and psychological symptoms in participants of female (vs male) sex (Table S3). Additionally, there was no clear evidence that sex moderated associations between confirmation of COVID-19 infection status and duration of symptoms (ongoing COVID-19 and post-COVID-19 syndrome; Table S4). However, results for the Long COVID composite, physical symptoms at 14–20 weeks, wellbeing, and both duration of symptoms variables were consistent with a possible clinically significant result that the study was too small to detect. There was no clear evidence that anxiety at the start of the pandemic moderated the associations between confirmation of COVID-19 infection status and the Long COVID composite, physical symptoms or psychological symptoms (Table S5). There was no clear evidence that those with (vs those without) anxiety at the start of the pandemic were more likely to report ongoing COVID-19 or post-COVID-19 syndrome (Table S6). However, the confidence intervals for all anxiety moderation analyses were consistent with the possibility of a clinically significant effect that our study was too small to detect.

### Associations between COVID-19 infection status and Long COVID outcomes (aim 3)

Descriptive statistics for Long COVID outcomes in those who report having had COVID-19 versus those who do not, are presented in Table S7, with tests of difference between groups. Associations between COVID-19 infection status and physical and psychological symptoms are reported in Table S8. There was no clear evidence that Long COVID composite scores differed between those who reported having COVID-19 and those who did not. There was strong evidence that having had COVID-19 (vs not) was associated with reporting fewer physical symptoms at 4–12 weeks (*B* −0.91, 95% CI −1.35 to −0.47, *p* < 0.001) and at 12–20 weeks (*B* −0.94, 95% CI −1.41 to −0.47, *p* < 0.001). Additionally, there was modest evidence that reporting having had COVID-19 (vs not) was associated with lower wellbeing (*B* −0.84, 95% CI −1.65 to −0.02, *p* = 0.004). The magnitude of effect, however, is unlikely to be clinically significant. There was no clear evidence of an association between COVID-19 infection status and depression or anxiety symptoms.

### Exploratory analyses

There was no clear evidence of moderation by sex on the relationship between confirmation of COVID-19 infection status and any of the Long COVID outcomes (Table S9). There was evidence that those with anxiety at the start of the pandemic (vs those without) showed a stronger association between previous COVID-19 infection and reporting fewer physical symptoms at both 4–12 weeks (*B* −1.12, 95% CI −2.12 to −0.12, *p* = 0.029) and 12–20 weeks (*B* −0.80, 95% CI −1.29 to −0.31, *p* = 0.001; Table S10). However, the magnitude of this effect is unlikely to be clinically significant. There was no clear evidence of moderation for Long COVID composite and psychological symptoms. For sex and anxiety moderation analyses, both the Long COVID composite and wellbeing results were too imprecise to exclude clinical significance.

### Sensitivity analyses

Complete case analyses are presented in Tables S11 to S19. There remained no clear evidence of association between confirmation of COVID-19 infection status and Long COVID outcomes (Tables S11 and S12) and no moderation effect by sex or anxiety (Tables S13–S16). The associations between COVID-19 infection status and both physical symptoms and wellbeing remained (Table S17). There was no moderating effect on these associations by sex (Table S18). Evidence of moderation by anxiety on the relationship between COVID-19 infection status on physical symptoms at 4–12 weeks remained; however, it did not remain for physical symptoms at 12–20 weeks (Table S19).

## Discussion

Using data from online questionnaires distributed to the ALSPAC cohort during the pandemic, we compared the presence of physical symptoms, psychological symptoms, duration of symptoms ascribed to COVID-19 and a Long COVID composite (composed of physical and psychological symptoms) between those with COVID-19 infection confirmed by positive test and suspected but not confirmed COVID-19 infection. Contrary to hypotheses 1 and 2, we found that confirmed (vs unconfirmed) COVID-19 infection was not associated with a greater incidence of Long COVID symptoms. However, the study was underpowered to exclude a clinically significant association with our Long COVID composite and wellbeing. Overall, the results suggest that confirmation of a COVID-19 diagnosis does not exert nocebo effects that contribute to the development of Long COVID. This contrasts with a cross-sectional study of French adults, which found that self-reported COVID-19 infection was associated with more persistent physical symptoms, whereas COVID-19 infection confirmed by serology was associated only with anosmia (Matta et al., 2022). However, among individuals with acute COVID-19 confirmed by polymerase chain reaction test, those who subsequently develop Long COVID are less likely to have formed an antibody response to COVID-19 (García-Abellán et al., 2021; Su et al., 2022). Therefore, concerns have been raised about drawing the conclusion that symptoms experienced by participants were not attributable to Long COVID using antibody testing (Alwan, 2022). In both Matta et al.’s work (2022) and ours, non-COVID-19 infections may have acted as a source of collider bias (Holmberg and Andersen, 2022). This is when a variable is influenced by both the exposure and outcome, and this variable is then controlled for within the study. Non-COVID-19 infections may contribute to both a belief in being infected with COVID-19 and experiencing Long COVID symptoms. By restricting the sample to those with a belief in being infected with COVID-19, the potential for a spurious association occurs.

Contrary to hypotheses 3 and 4, there was no clear evidence of moderation by sex or anxiety. This contrasts with a previous study which found that certainty of being infected with COVID-19 was associated with reporting more COVID-19 symptoms, and that this effect was greater in participants with anxiety (Daniali and Flaten, 2022). The authors excluded those who reported having had a positive test, whilst in our study we examined the impact of a positive test result and so other sources of nocebo effects may be responsible for the association they found. Additionally, their study examined the acute viral period. It is possible that any existing nocebo effects from positive diagnosis in the present study did not persist into the Long COVID period (>4 weeks).

Finally, contrary to hypothesis 6, those who reported a previous COVID-19 infection reported fewer physical symptoms than those who did not. This association was stronger amongst those who reported anxiety at the start of the pandemic. Other outcomes were similar between the two groups. Thompson et al. (2022) also found that when quantifying Long COVID using symptom reporting over time, there was a substantial prevalence of Long COVID in both those with and without previous COVID-19 infection. However, in their study the prevalence remained greater among individuals who reported COVID-19 infection than in uninfected people. Several factors may have affected the number of symptoms reported by non-COVID-19 participants. We recorded the symptoms they reported during December 2020, a time that England was under quarantine measures to prevent the spread of COVID-19, which may have had its own effects on the symptoms experienced by participants. This was additionally the peak of the rate of coronavirus infections in the UK during the study period (Public Health England, 2022), and it is possible a proportion of this group may have been affected by undiagnosed COVID-19 infection. Furthermore, non-infectious conditions that were not controlled for may have caused the symptoms that we recorded.

Our study found no clear evidence of a nocebo effect on the development of Long COVID, adding to our understanding of the pathophysiology of this poorly understood condition and providing reassurance to clinicians interacting with patients with COVID-19. There have historically been concerns about nocebo effects arising when diagnosing conditions such as ME/CFS (Huibers and Wessely, 2006). Whilst our study focused on the effect of acute COVID-19 infection diagnosis, rather than Long COVID diagnosis, it lends evidence against this argument. A strength of our study is the inclusion of data on both symptoms participants ascribed to COVID-19 (i.e. our ‘duration of symptoms’ variable) and also physical and psychological symptoms irrespective of perceived cause. This increases the sensitivity, by capturing both those who believe they have Long COVID and those who may be experiencing Long COVID unknowingly. A second strength is our method of quantifying Long COVID symptoms. Many Long COVID symptoms are common and non-specific, and some studies may overestimate the prevalence of Long COVID by counting any participant reporting symptoms as experiencing Long COVID. To negate this, we compared total number of symptoms using continuous variables rather than stipulating a cut-off. Our study confirms that these symptoms are common amongst individuals who have not had COVID-19 and highlights the importance of including a control group.

Our study has several limitations. First, our temporal categorisation of symptoms may have led to missing cases of Long COVID. The months for which symptoms were recorded as occurring in the ‘ongoing COVID’ period started a minimum of 4 weeks after infection date to avoid measuring symptoms experienced in the acute COVID-19 illness period but could have started up to 8 weeks after. Similarly, the ‘post-COVID-19’ period could have started between 12 and 16 weeks after infection date. Some participants may have had symptoms for more than 4 weeks which had ceased prior to the month we measured. However, the majority of respondents to an ONS survey who had self-reported Long COVID reported symptoms lasting over 12 weeks (ONS, 2023) and therefore it is unlikely that we have missed a substantial proportion of Long COVID.

Second, we did not measure symptom severity, symptom frequency or impact on daily functioning. It is possible that Long COVID represents an increased severity or frequency of symptoms, which would have been missed in our outcome measures. However, the reported duration of symptoms participants attributed to COVID-19 was also similar between those with confirmed and unconfirmed COVID-19, which provides reassurance that there was not an unmeasured difference in Long COVID prevalence between the two groups. Furthermore, the current study did not include markers of severity of the original COVID illness, treatment received for acute COVID-19 infection or COVID-19 vaccination status.

Third, we decided that a minimum clinically important difference would be an increase or decrease in the number of symptoms by two or more for continuous outcomes. This is limited without measures of severity, as a single but severe new symptom would likely be significant for both patients and clinicians. Future research would benefit from patient and clinician input in defining the minimum clinically important difference. Fourth, some analyses had low sample sizes and may have lacked power to detect an effect. Finally, it is possible that missing data was non-random, as those with Long COVID may have been less likely to complete questionnaires, and therefore biased our study to the null.

To reconcile the results of the current study with other work demonstrating a potential nocebo effect, future work is needed to examine the impact of other potential sources of nocebo on the experience of Long COVID symptoms. Given the lack of nocebo in the current study, future work should consider other mechanisms contributing to developing Long COVID. Future research is also needed to understand the source of symptoms reported by groups not affected by COVID-19 infection and to understand the possible contribution of quarantine measures to people’s experience of physical symptoms.

## Conclusions

Confirmation of COVID-19 infection by positive test was not associated with increased reporting of Long COVID symptoms. Furthermore, there was no clear evidence of moderation by either sex or anxiety. There was no evidence that a COVID-19 diagnosis exerted nocebo effects contributing to the aetiology of Long COVID.

## Supplemental Material

sj-docx-1-hpq-10.1177_13591053241228711 – Supplemental material for Confirmation of COVID-19 infection status and reporting of Long COVID symptoms in a population-based birth cohort: No evidence of a nocebo effect Supplemental material, sj-docx-1-hpq-10.1177_13591053241228711 for Confirmation of COVID-19 infection status and reporting of Long COVID symptoms in a population-based birth cohort: No evidence of a nocebo effect by Catherine IA Macleod-Hall, Marcus R Munafò and Maddy L Dyer in Journal of Health Psychology
