# Supplementary material for: Confirmation of COVID-19 infection status and reporting of Long COVID symptoms in a population-based birth cohort: No evidence of a nocebo effect
Source: J Health Psychol. 2024 Jan 25;29(6):581–94. doi: 10.1177/13591053241228711 (PMC11144352; doi:10.1177/13591053241228711)
Supplement: sj-docx-1-hpq-10.1177_13591053241228711 – Supplemental material for Confirmation of COVID-19 infection status and reporting of Long COVID symptoms in a population-based birth cohort: No evidence of a nocebo effect [file sj-docx-1-hpq-10.1177_13591053241228711.docx]

**Supplementary Table S1. Data dictionary**

| **Exposure Variables** | |
| --- | --- |
| All four questionnaires ask: ‘do you think you have had COVID?’ Possible answers are: ‘yes, confirmed by a positive test’, ‘yes, suspected by a doctor but not tested’, ‘yes, my own suspicions’, or ‘no’. | |
| Confirmation of COVID-19 infection status | ‘Confirmed COVID-19’ = ‘yes, confirmed by a positive test’  ‘Unconfirmed COVID-19’ = ‘yes, suspected by a doctor but not tested’ or ‘yes, my own suspicions’.  Participants who responded ‘no’ excluded.  Categories ‘yes, confirmed by a positive test’ and ‘yes, suspected by a doctor but not tested’ are combined in released data for questionnaire 1 due to low cell counts. We therefore excluded questionnaire 1 responses from this variable, as a deviation from our protocol.  Binary: 0 = ‘Unconfirmed COVID-19’, 1 = ‘Confirmed COVID-19’. |
| COVID-19 Infection Status | ‘Reported COVID-19’ = ‘yes, confirmed by a positive test’, ‘yes, suspected by a doctor but not tested’, ‘yes, my own suspicions’.  ‘No COVID-19’ = ‘no’.  Binary: 0 = ‘No COVID-19’, 1 = ‘Reported COVID-19’. |
| **Outcome Variables** | |
| Long COVID composite | Continuous variable: summed physical symptoms in the relevant timeframe and scoring 1 for each of the following: Short Mood and Feelings Questionnaire Score (SMFQ) ≥12, Generalised Anxiety Disorder-7 questionnaire (GAD-7) ≥ and Warwick-Edinburgh Mental Well-being Scale (WEMWS) ≤40.  COVID-19 participants: physical symptoms = a sum of the values of the variables ‘physical symptoms at 4 – 12 weeks’ and ‘physical symptoms at 12 – 20 weeks’.  Non-COVID-19 participants: physical symptoms = the sum of those reported in September to December 2020. If they had not answered for the month of December, the months of August to November 2020 were summed. If they had not completed questionnaire 4, July to October 2020 symptoms were summed or if they had only completed questionnaire 1, symptoms from January to April 2020 were summed. |
| *Physical symptoms* | |
| Physical symptoms self-reported each month between October 2019 and March 2021 in questionnaires 1, 3 and 4.  24 possible symptoms: decrease in appetite, nausea and/or vomiting, diarrhoea, abdominal pain, runny nose, sneezing, sore eyes, loss of sense of smell or taste, sore throat, hoarse voice, headache, numbness or tingling, dizziness, cough, tightness in the chest, shortness of breath, chest pain, fever, chills, difficulty sleeping, feeling more tired than normal, severe fatigue, feeling of heaviness in arms or legs, achy muscles.  The following were excluded as they were not asked about in every questionnaire: painful joints, tender neck, hair loss, painful sores or blisters, sudden face swelling, and red or itchy skin.  We summed symptoms across the two months (i.e., possible total of 48) rather than scoring one if a symptom was reported in either month (i.e., possible total of 24). This is because those with symptoms that recurred or persisted over two months would have higher scores, better reflecting long COVID, which can be either a relapsing/recurring or continuous syndrome (BMJ, 2020). | |
| Physical symptoms at 4 – 12 weeks | Continuous variable: summed physical symptoms in the relevant timeframe.  COVID-19 participants: If a participant reported having had COVID-19 in month one, we summed physical symptoms reported in month three and four.  Non-COVID-19 participants: We summed data from November and December 2021, as these were the months closest to the peak rate of coronavirus infections in the UK (Public Health England, 2022) with an adequate response rate. Questions about January and February had a lower response rate, due to participants completing the survey before these dates occurred. We also used data from October and November for questionnaire 4 respondents with missing data for December, to ensure that all participants had data for two months counted. For those who did not complete questionnaire 4, we used data from the latest questionnaire they completed; we used the latest two months surveyed, rather than the earliest two months as initially planned, so that all months fell within the pandemic period (i.e., after March 2020). |
| Physical symptoms at 12 - 20 weeks | Continuous variable: summed physical symptoms in the relevant timeframe.  COVID-19 participants: If a participant reported having had COVID-19 in month one, we summed physical symptoms reported in month five and six.  Non-COVID-19 participants: as above. |
| *Psychological symptoms* | |
| Questionnaires 1, 2 and 4 assessed depression, anxiety, and wellbeing within the previous 2 weeks.  COVID-19 participants: the responses from the questionnaire that was completed more than 4 weeks after their COVID-19 illness start date was used.  Non-COVID-19 participants: their response from questionnaire 4 was used as it was closest to the peak rate of infections in the UK, or the latest questionnaire they completed. | |
| Depression | Continuous variable: total score on SMFQ, a 13-item scale with a possible range 0-26.  Higher scores indicate greater severity of depression symptoms. |
| Anxiety | Continuous variable: total score on GAD-7, a 7-item scale with a possible range 0-21.  Higher scores indicate greater severity of anxiety symptoms. |
| Wellbeing | Continuous variable: total score on WEMWS, a 14-item scale with a possible range 14-70.  Higher scores indicate greater wellbeing. |
| *Duration of symptoms* | |
| In questionnaire 4, participants are asked about the total overall length of symptoms they believe are related to COVID-19. Possible answers included: ‘1 day – 2 weeks’, ‘2 - 4 weeks’, ‘4 - 12 weeks’ and ‘12+ weeks’. | |
| Ongoing COVID-19 | ‘Acute COVID-19’ = symptoms 1 day – 2 weeks or 2 - 4 weeks in length.  ‘Ongoing COVID-19’ = symptoms 4 – 12 weeks or 12+ weeks in length.  Binary variable: 0 = ‘acute COVID’, 1 = ‘ongoing COVID-19’. |
| Post-COVID-19 | ‘Acute or ongoing COVID-19’ = symptoms 1 day – 2 weeks or 2 - 4 weeks or 4 – 12 weeks in length.  ‘Post-COVID-19 syndrome’ = symptoms 12+ weeks in length.  Binary variable: 0 = ‘Acute or ongoing COVID-19’, 1 = ‘post-COVID-19 syndrome’. |
| **Moderator variables and covariates** | |
| Sex | Supplied from previous ALSPAC data.  Binary: 0 = ‘Male’, 1 = ‘Female’. |
| Anxiety at the start of the pandemic | Reported on questionnaire 1 at the start of the pandemic.  Binary: 0 = ‘No’, 1 = ‘Yes’. |
| Age | Age at the earliest questionnaire they completed.  Categorical variable: 0 = ≤35, 1 = 36-59, 2 ≥60.  Categorised this way to separate |
| Body mass index | Supplied from most recent ALSPAC clinic visit.  Binary variable: 0 = ≤25, 1 = 26 or more.  Dichotomised at 25 due to increased risk of severe COVID-19 illness in overweight or obese people. |
| White ethnic group | Supplied from previous ALSPAC data.  Binary variable: 0 = ‘White’, 1 = ‘Non-White’.  Potential responses to self-reported ethnicity: White, Black/Caribbean, Black/African, Black/other, Indian, Pakistani, Bangladeshi, Chinese, Other Specified.  Dichotomised as white versus non-white as white was the largest subgroup. |
| Comorbidities at the start of the pandemic | Reported on questionnaire 1 at the start of the pandemic.  One or more of: diabetes (Type I or II), heart disease or heart problems, hypertension, kidney disease, liver disease, anaemia, asthma, other lung conditions, cancer, and weakened immune system/reduced ability to fight infections.  Binary: 0 = ‘No’, 1 = ‘Yes’. |

**Supplementary Table S2. Study sample size flow diagram.**


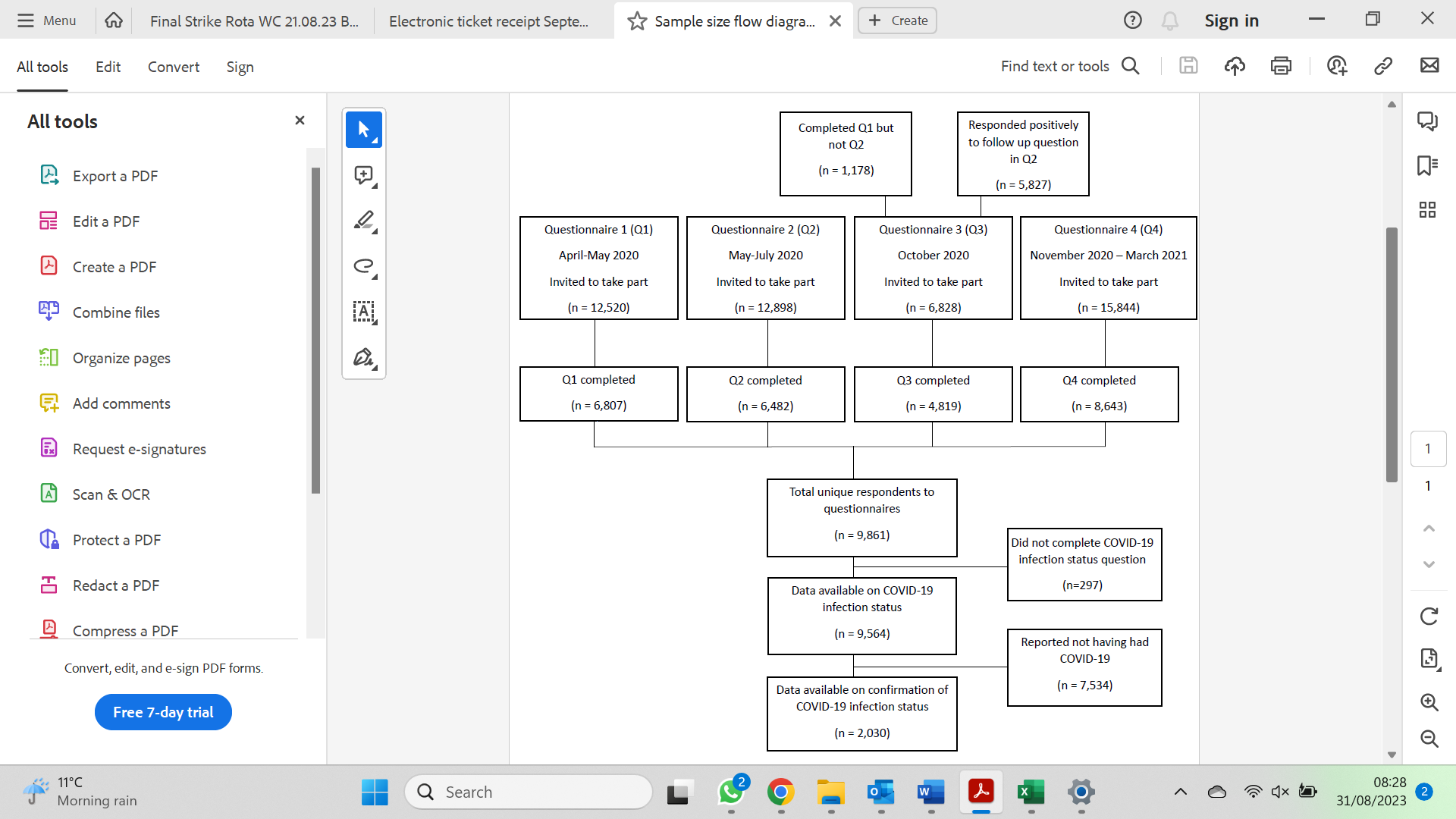


**Supplementary Table S3. Moderation by sex on the relationship between confirmation of COVID-19 infection status and physical and psychological symptoms**

|  | | n | B (95% CI) | *p*-value |
| --- | --- | --- | --- | --- |
| **Long COVID Composite** | | | | |
| Sex | Male | 319 | -0.03 (-2.66 to 2.60) | .982 |
|  | Female | 132 | -1.20 (-4.24 to 1.84) | .435 |
| Interaction | Model A | 453 | -1.17 (-5.60 to 3.25) | .602 |
|  | Model B | 451 | -0.39 (-2.44 to 1.65) | .704 |
|  | Model C | 341 | -2.96 (-8.16 to 2.24)* | .264 |
| **Physical Symptoms 4 – 12 Weeks** | | | | |
| Sex | Male | 534 | -1.04 (-1.95 to -0.13) | .025 |
|  | Female | 222 | -0.26 (-1.38 to 0.86) | .649 |
| Interaction | Model A | 756 | 0.78 (-0.79 to 2.35) | .330 |
|  | Model B | 756 | -0.81 (-1.53 to -0.09) | .028 |
|  | Model C | 523 | 0.36 (-1.80 to 0.30) | .163 |
| **Physical Symptoms 12-20 Weeks** | | | | |
| Sex | Male | 385 | -0.12 (-1.21 to 0.97) | .831 |
|  | Female | 158 | -1.02 (-2.42 to 0.38) | .152 |
| Interaction | Model A | 543 | -0.12 (-1.15 to 0.92) | .823 |
|  | Model B | 543 | -0.39 (-1.25 to 0.48) | .385 |
|  | Model C | 403 | -0.89 (-3.23 to 1.43)* | .451 |
| **Depression** | | | | |
| Sex | Male | 467 | 0.86 (-0.37 to 2.09) | .169 |
|  | Female | 220 | 0.30 (-1.17 to 1.77) | .687 |
| Interaction | Model A | 687 | -0.56 (-2.60 to 1.48) | .590 |
|  | Model B | 687 | 0.68 (-0.28 to 1.64) | .163 |
|  | Model C | 477 | 0.90 (-1.85 to 0.70) | .376 |
| **Anxiety** | | | | |
| Sex | Male | 472 | 0.42 (-0.70 to 1.55) | .459 |
|  | Female | 226 | -0.41 (-1.80 to 0.94) | .551 |
| Interaction | Model A | 698 | -0.83 (-2.70 to 1.03) | .381 |
|  | Model B | 698 | 0.15 (-0.72 to 1.03) | .732 |
|  | Model C | 484 | -0.23 (-1.42 to 0.97) | .708 |
| **Wellbeing** | | | | |
| Sex | Male | 474 | 0.27 (-1.68 to 2.22) | .785 |
|  | Female | 220 | 0.79 (-1.97 to 3.55) | .572 |
| Interaction | Model A | 694 | 0.52 (-2.87 to 3.91) | .783 |
|  | Model B | 694 | 0.44 (-1.15 to 2.03) | .586 |
|  | Model C | 482 | 3.75 (-1.93 to 9.43)* | .195 |

Notes: 95% CI: 95% confidence intervals. B: unstandardised coefficients. An asterisk (*) denotes an adjusted model for which the confidence intervals are consistent with a clinically significant result that our study was too small to detect.

Confirmed COVID-19, coded ‘1’, is confirmed by any test; unconfirmed COVID-19, coded ‘0’, is suspected by participant or doctor but not tested. Long COVID composite is a composite of physical symptoms reported at 4-20 weeks after onset of COVID-19 infection, and psychological symptoms. Depression symptoms measured using the Short Mood and Feelings Questionnaire (SMFQ), anxiety symptoms measured using the Generalised Anxiety Disorder-7 questionnaire (GAD-7) and wellbeing measured using the Warwick-Edinburgh Mental Well-being Scale (WEMWS).

Linear regressions. Sex: unadjusted associations between confirmation of COVID-19 infection status and duration of symptoms, stratified by sex (male vs female). Model A: unadjusted interaction of confirmation of having had COVID-19 x sex on physical and psychological symptoms. Model B: association between confirmation of having had COVID-19 and physical and psychological symptoms, adjusted for sex. Model C: interaction of confirmation of having had COVID-19 x sex on physical and psychological symptoms, adjusted for age, body mass index, ethnicity, anxiety, and comorbidities at the start of the pandemic. Likelihood ratios comparing model A and model B: long COVID composite – χ^2^ 0.27, *p* =.600; physical symptoms at 4-12 weeks – χ^2^ 0.96, *p* =.328; physical symptoms 12-20 weeks - χ^2^ 0.87, p=.350; depression – χ^2^ 0.29, *p* =.589; anxiety - χ^2^ 0.77, *p* =.380; wellbeing - χ^2^ 0.09, *p* =.763.

**Supplementary Table S4. Moderation by sex on the relationship between confirmation of COVID-19 infection status and duration of symptoms**

|  | | n | Odds ratio (95% CI) | *p*-value |
| --- | --- | --- | --- | --- |
| **Ongoing COVID-19** | | | | |
| Sex | Male | 754 | 0.82 (0.57 to 1.18) | .282 |
|  | Female | 392 | 0.65 (0.36 to 1.18) | .157 |
| Interaction | Model A | 1,146 | 0.79 (0.51 to 1.59) | .507 |
|  | Model B | 1,146 | 0.77 (0.57 to 1.05) | .096 |
|  | Model C | 638 | 0.50 (0.17 to 1.46)* | .206 |
| **Post-COVID-19 Syndrome** | | | | |
| Sex | Male | 754 | 0.65 (0.39 to 1.08) | .097 |
|  | Female | 392 | 0.70 (0.28 to 1.76) | .451 |
| Interaction | Model A | 1,146 | 1.08 (0.38 to 3.10) | .097 |
|  | Model B | 1,146 | 0.66 (0.42 to 1.03) | .069 |
|  | Model C | 638 | 1.17 (0.37 to 1.46)* | .833 |

Notes: 95% CI: 95% confidence intervals. An asterisk (*) denotes an adjusted model for which the confidence intervals are consistent with a clinically significant result that our study was too small to detect.

Confirmed COVID-19, coded ‘1’, is confirmed by any test; unconfirmed COVID-19, coded ‘0’, is suspected by participant or doctor but not tested. Ongoing COVID-19: symptoms 4 weeks or more after the onset of the original COVID-19 infection. Post-COVID-19 syndrome: symptoms 12 weeks or more after the onset of the original COVID-19 infection.

Logistic regressions. Sex: unadjusted associations between confirmation of COVID-19 infection status and duration of symptoms, stratified by sex (male vs female). Model A: unadjusted interaction of confirmation of having had COVID-19 x sex on duration of symptoms. Model B: association between confirmation of having had COVID-19 and duration of symptoms, adjusted for sex. Model C: interaction of confirmation of having had COVID-19 x sex on duration of symptoms, adjusted for age, body mass index, ethnicity, anxiety, and comorbidities at the start of the pandemic. Likelihood ratios comparing model A and model B: ongoing COVID-19 – χ^2^ 0.45, *p* = .504; post-COVID-19 syndrome – χ^2^  0.02, *p* = .885.

**Supplementary Table S5. Moderation by anxiety on the relationship between confirmation of COVID-19 infection status and physical and psychological symptoms**

|  | | n | B (95% CI) | *p*-value |
| --- | --- | --- | --- | --- |
| **Long COVID Composite** | | | | |
| Anxiety | No | 299 | -0.78 (-2.94 to 1.39) | .480 |
|  | Yes | 76 | 3.00 (-5.78 to 11.77) | .499 |
| Interaction | Model A | 375 | 3.77 (-2.51 to 10.05) | .562 |
|  | Model B | 375 | -0.11 (-2.51 to 2.28) | .926 |
|  | Model C | 341 | -1.21 (-8.22 to 5.80)* | .735 |
| **Physical Symptoms 4 – 12 Weeks** | | | | |
| Anxiety | No | 459 | -0.61 (-1.44 to 0.21) | .146 |
|  | Yes | 115 | -1.38 (-4.36 to 1.60) | .360 |
| Interaction | Model A | 574 | -0.77 (-3.04 to 1.51) | .509 |
|  | Model B | 574 | -0.75 (-1.62 to 0.12) | .091 |
|  | Model C | 523 | -2.02 (-4.43 to 0.37)* | .098 |
| **Physical Symptoms 12-20 Weeks** | | | | |
| Anxiety | No | 359 | -0.12 (-1.06 to 0.82) | .802 |
|  | Yes | 85 | 1.12 (-2.92 to 5.15) | .583 |
| Interaction | Model A | 444 | 1.24 (-1.66 to 4.13) | .402 |
|  | Model B | 444 | 0.06 (-0.96 to 1.09) | .906 |
|  | Model C | 403 | -1.25 (-4.58 to 2.07)* | .459 |
| **Depression** | | | | |
| Anxiety | No | 422 | -0.43 (-1.49 to 0.63) | .429 |
|  | Yes | 102 | 4.07 (0.46 to 7.68) | .027 |
| Interaction | Model A | 524 | 4.50 (1.63 to 7.36) | .002 |
|  | Model B | 524 | 0.35 (-0.74 to 1.44) | .532 |
|  | Model C | 477 | 1.10 (-1.91 to 4.10)* | .473 |
| **Anxiety** | | | | |
| Anxiety | No | 428 | -0.04 (-1.06 to 0.10) | .933 |
|  | Yes | 103 | 1.20 (-1.01 to 4.38) | .467 |
| Interaction | Model A | 531 | 1.22 (-1.48 to 3.92) | .376 |
|  | Model B | 532 | 0.16 (-0.85 to 1.17) | .753 |
|  | Model C | 484 | -2.19 (-5.03 to 0.64)* | .129 |
| **Wellbeing** | | | | |
| Anxiety | No | 427 | 1.23 (-0.83 to 3.29) | .242 |
|  | Yes | 102 | -0.11 (-5.14 to 4.92) | .966 |
| Interaction | Model A | 529 | -1.37 (-6.44 to 3.77) | .607 |
|  | Model B | 529 | 1.00 (-0.90 to 2.91) | .302 |
|  | Model C | 482 | 3.75 (-1.93 to 9.43)* | .195 |

Notes: 95% CI: 95% confidence intervals. B: Unstandardised coefficients. An asterisk (*) denotes an adjusted model for which the confidence intervals are consistent with a clinically significant result that our study was too small to detect.

Confirmed COVID-19, coded ‘1’, is confirmed by any test; unconfirmed COVID-19, coded ‘0’, is suspected by participant or doctor but not tested. Long COVID composite is a composite of physical symptoms reported at 4-20 weeks after onset of COVID-19 infection, and psychological symptoms. Depression symptoms measured using the Short Mood and Feelings Questionnaire (SMFQ), anxiety symptoms measured using the Generalised Anxiety Disorder-7 questionnaire (GAD-7) and wellbeing measured using the Warwick-Edinburgh Mental Well-being Scale (WEMWS).

Linear regressions. Anxiety: unadjusted associations between confirmation of COVID-19 infection status and duration of symptoms, stratified by presence of anxiety (no vs yes). Model A: unadjusted interaction of confirmation of having had COVID-19 x anxiety on physical and psychological symptoms. Model B: association between confirmation of having had COVID-19 and physical and psychological symptoms, adjusted for anxiety. Model C: interaction of confirmation of having had COVID-19 x anxiety on physical and psychological symptoms, adjusted for age, sex, body mass index, ethnicity, and comorbidities at the start of the pandemic. Likelihood ratios comparing model A and model B: long COVID composite – χ^2^ 1.41, *p* =.235; physical symptoms 4 - 12 weeks – χ^2^ 0.44, *p* =.507; physical symptoms 12 – 20 weeks – χ^2^ 0.71, *p* =.400; depression – χ^2^ 9.47, *p* =.002; anxiety - χ^2^ 0.79, *p* =.373; wellbeing - χ^2^ 0.27, *p* =.606.

**Supplementary Table S6. Moderation by anxiety on the relationship between confirmation of COVID-19 infection status and duration of symptoms**

|  | | n | Odds ratio (95% CI) | *p*-value |
| --- | --- | --- | --- | --- |
| **Ongoing COVID-19** | | | | |
| Anxiety | No | 567 | 0.71 (0.45 to 1.12) | .139 |
|  | Yes | 135 | 1.70 (0.72 to 4.05) | .228 |
| Interaction | Model A | 702 | 2.40 (0.90 to 6.39) | .079 |
|  | Model B | 702 | 0.85 (0.57 to 1.26) | .416 |
|  | Model C | 638 | 2.13 (0.70 to 6.44)* | .182 |
| **Post-COVID-19 Syndrome** | | | | |
| Anxiety | No | 567 | 0.56 (0.29 to 1.11) | .099 |
|  | Yes | 135 | 2.04 (0.69 to 6.02) | .195 |
| Interaction | Model A | 702 | 3.62 (1.01 to 13.00) | .048 |
|  | Model B | 702 | 0.77 (0.44 to 1.36) | .364 |
|  | Model C | 638 | 3.70 (0.92 to 14.86)* | .065 |

Notes: 95% CI: 95% confidence intervals. An asterisk (*) denotes an adjusted model for which the confidence intervals are consistent with a clinically significant result that our study was too small to detect.

Confirmed COVID-19, coded ‘1’, is confirmed by any test; unconfirmed COVID-19, coded ‘0’, is suspected by participant or doctor but not tested. Ongoing COVID-19: symptoms 4 weeks or more after the onset of the original COVID-19 infection. Post-COVID-19 syndrome: symptoms 12 weeks or more after the onset of the original COVID-19 infection.

Logistic regressions. Anxiety: unadjusted associations between confirmation of COVID-19 infection status and duration of symptoms, stratified by presence of anxiety (no vs yes). Model A: unadjusted interaction of confirmation of having had COVID-19 x anxiety on duration of symptoms. Model B: association between confirmation of having had COVID-19 and duration of symptoms, adjusted for anxiety. Model C: interaction of confirmation of having had COVID-19 x anxiety on duration of symptoms, adjusted for age, sex, body mass index, ethnicity and comorbidities at the start of the pandemic. Likelihood ratios comparing model A and model B: ongoing COVID-19 – χ^2^ 3.01, *p* = .082; post-COVID-19 syndrome – χ^2^ 3.72, *p* = .054.

**Supplementary Table S7. Self-reported COVID-19 infection status and physical and psychological symptoms**

|  | *n* | No COVID-19 | Reported COVID-19 | *p*-value |
| --- | --- | --- | --- | --- |
| Long COVID Composite, Mean (SD) | 4,009 | 7.3 (8.2) | 6.99 (9.7) | .434 |
| **Physical Symptoms, Mean (SD)** | | | | |
| Physical Symptoms at 4 – 12 Weeks | 8,134 | 3.9 (4.9) | 2.9 (4.9) | <.001 |
| Physical symptoms at 12 – 20 Weeks | 7,921 | 3.9 (4.9) | 2.7 (4.5) | <.001 |
| **Psychological Symptoms, Mean (SD)** | | | | |
| Depression | 7,781 | 4.8 (5.3) | 5.3 (5.4) | .019 |
| Anxiety | 7,860 | 5.2 (5.1) | 5.4 (5.0) | .307 |
| Wellbeing | 7,790 | 46.7 (9.4) | 45.5 (9.0) | .001 |

Notes: SD: standard deviation

Self-reported COVID-19 infection, coded ‘1’, is confirmed by test or suspected by participant or doctor but not tested. No COVID-19, coded ‘0’, applied to participants reporting not having had COVID-19. Long COVID composite is a composite of physical symptoms reported at 4-20 weeks after onset of COVID-19 infection, and psychological symptoms. Depression symptoms measured using the Short Mood and Feelings Questionnaire (SMFQ), anxiety symptoms measured using the Generalised Anxiety Disorder-7 questionnaire (GAD-7) and wellbeing measured using the Warwick-Edinburgh Mental Well-being Scale (WEMWS). T-tests were used to assess differences between groups for continuous outcomes, and chi-squared tests for dichotomous outcomes.

**Supplementary Table S8. Associations between self-reported COVID-19 infection status and reported physical and psychological symptoms**

|  | Unadjusted | | | Adjusted | | |
| --- | --- | --- | --- | --- | --- | --- |
|  | *n* | B (95% CI) | *p*-value | *n* | B (95% CI) | *p*-value |
| Long COVID Composite | 4,009 | -0.33 (-1.26 to 0.61) | .493 | 3,271 | -0.73 (-1.79 to 0.33) | .178 |
| **Physical Symptoms** | | | | | | |
| Physical Symptoms at 4 – 12 Weeks | 8,134 | -0.92 (-1.28 to -0.55) | <.001 | 5,121 | -0.91 (-1.35 to -0.47) | <.001 |
| Physical Symptoms at 12 – 20 Weeks | 7,921 | -1.13 (-1.53 to -0.73) | <.001 | 5,001 | -0.94 (-1.41 to -0.47) | <.001 |
| **Psychological Symptoms** | | | | | | |
| Depression | 7,781 | 0.50 (0.08 to 0.92) | 0.021 | 4,837 | -0.08 (-0.51 to 0.35) | .712 |
| Anxiety | 7,860 | 0.20 (-0.18 to 0.600 | 0.299 | 4,883 | -0.17 (-0.58 to 0.24) | .411 |
| Wellbeing | 7,790 | -1.20 (-1.91 to -0.49) | 0.001 | 4,845 | -0.84 (-1.65 to -0.02) | .004 |

Notes: 95% CI: 95% confidence intervals. B: unstandardised coefficients.

Linear regressions. Adjusted analyses are adjusted for age, sex, body mass index, ethnicity, anxiety, and comorbidities at the start of the pandemic. Self-reported COVID-19 infection, coded ‘1’, is confirmed by test or suspected by participant or doctor but not tested. No COVID-19, coded ‘0’, applied to participants reporting not having had COVID-19. Long COVID composite is a composite of physical symptoms reported at 4-20 weeks after onset of COVID-19 infection, and psychological symptoms. Depression symptoms measured using the Short Mood and Feelings Questionnaire (SMFQ), anxiety symptoms measured using the Generalised Anxiety Disorder-7 questionnaire (GAD-7) and wellbeing measured using the Warwick-Edinburgh Mental Well-being Scale (WEMWS).

**Supplementary Table S9. Moderation by sex on the relationship between self-reported COVID-19 infection status and physical and psychological symptoms**

|  |  | *n* | B (95% CI) | *p*-value |
| --- | --- | --- | --- | --- |
| **Long COVID Composite** | | | | |
| Sex | Male | 2,887 | -0.31 (-1.34 to 0.72) | .552 |
|  | Female | 1,122 | -0.25 (-1.46 to 0.97) | .692 |
| Interaction | Model A | 4,009 | 0.07 (-1.73 to 1.86) | .942 |
|  | Model B | 4,009 | -0.29 (-1.11 to 0.52) | .480 |
|  | Model C | 3,271 | -0.12 (-2.03 to 1.79)* | .903 |
| **Physical Symptoms 4 – 12 Weeks** | | | | |
| Sex | Male | 5,619 | -1.02 (-1.47 to -0.57) | <.001 |
|  | Female | 2,515 | -0.72 (-1.31 to -0.14) | .016 |
| Interaction | Model A | 8,134 | 0.30 (-0.49 to 1.09) | .461 |
|  | Model B | 8,134 | -0.93 (-1.30 to -0.57) | <.001 |
|  | Model C | 5,121 | 0.15 (-0.75 to 1.04) | .749 |
| **Physical Symptoms 12-20 Weeks** | | | | |
| Sex | Male | 5,470 | -1.20 (-1.72 to -0.68) | <.001 |
|  | Female | 2,451 | -1.03 (-1.72 to -0.35) | .003 |
| Interaction | Model A | 7,921 | 0.17 (-0.75 to 1.09) | .717 |
|  | Model B | 7,921 | -1.15 (-1.57 to -0.73) | <.001 |
|  | Model C | 5,001 | 0.12 (-0.87 to 1.10) | .811 |
| **Depression** | | | | |
| Sex | Male | 5,351 | 0.47 (-0.06 to 0.98) | .079 |
|  | Female | 2,430 | 0.60 (-0.06 to 1.25) | .073 |
| Interaction | Model A | 7,781 | 0.12 (-0.77 to 1.02) | .785 |
|  | Model B | 7,781 | 0.51 (0.10 to 0.93) | .016 |
|  | Model C | 4,837 | -0.18 (-1.11 to 0.75) | .704 |
| **Anxiety** | | | | |
| Sex | Male | 5,390 | 0.06 (-0.44 to 0.56) | .817 |
|  | Female | 2,470 | 0.57 (-0.03 to 1.18) | .064 |
| Interaction | Model A | 7,860 | 0.51 (-0.33 to 1.35) | .230 |
|  | Model B | 7,860 | 0.23 (-0.17 to 0.62) | .226 |
|  | Model C | 4,883 | 0.28 (-0.60 to 1.17) | .530 |
| **Wellbeing** | | | | |
| Sex | Male | 5,360 | -0.81 (-1.69 to 0.08) | .075 |
|  | Female | 2,430 | -2.09 (-3.36 to -0.82) | .001 |
| Interaction | Model A | 7,790 | -1.28 (-2.85 to 0.28) | .108 |
|  | Model B | 7,790 | -1.21 (-1.94 to -0.48) | .001 |
|  | Model C | 4,845 | 0.17 (-1.92 to 2.26)* | .874 |

Notes: 95% CI: 95% confidence intervals. B: unstandardised coefficients. An asterisk (*) denotes an adjusted model for which the confidence intervals are consistent with a clinically significant result that our study was too small to detect.

Self-reported COVID-19 infection, coded ‘1’, is confirmed by test or suspected by participant or doctor but not tested. No COVID-19, coded ‘0’, applied to participants reporting not having had COVID-19. Long COVID composite is a composite of physical symptoms reported at 4-20 weeks after onset of COVID-19 infection, and psychological symptoms. Depression symptoms measured using the Short Mood and Feelings Questionnaire (SMFQ), anxiety symptoms measured using the Generalised Anxiety Disorder-7 questionnaire (GAD-7) and wellbeing measured using the Warwick-Edinburgh Mental Well-being Scale (WEMWS).

Linear regressions. Sex: unadjusted associations between confirmation of COVID-19 infection status and duration of symptoms, stratified by sex (male vs female). Model A: unadjusted interaction of reporting having had COVID-19 x sex on physical and psychological symptoms. Model B: association between reporting having had COVID-19 and physical and psychological symptoms, adjusted for sex. Model C: interaction of reporting having had COVID-19 x sex on physical and psychological symptoms, adjusted for age, body mass index, ethnicity, anxiety, and comorbidities at the start of the pandemic. Likelihood ratios comparing model A and model B: long COVID composite - χ^2^ 0.01, *p* =.941; physical symptoms 4-12 weeks – χ^2^ 0.54, *p* =.461; physical symptoms at 12-20 weeks – χ^2^ 0.13*, p* =.717; depression – χ^2^ 0.07, *p* =.786; anxiety – χ^2^ 1.44, *p* =.230; wellbeing – χ^2^ 2.59, *p* =108.

**Supplementary Table S10. Moderation by anxiety on the relationship between self-reported COVID-19 infection status and physical and psychological symptoms**

|  |  | *n* | B (95% CI) | *p*-value |
| --- | --- | --- | --- | --- |
| **Long COVID Composite** | | | | |
| Anxiety | No | 2,978 | -0.75 (-2.87 to 1.37) | .487 |
|  | Yes | 618 | -1.12 (-3.93 to 1.68) | .431 |
| Interaction | Model A | 3,596 | -0.37 (-1.32 to 0.57) | .440 |
|  | Model B | 3,596 | -0.52 (-1.37 to 0.32) | .226 |
|  | Model C | 3,271 | -0.92 (-3.11 to 1.26)* | .408 |
| **Physical Symptoms 4 – 12 Weeks** | | | | |
| Anxiety | No | 4,584 | -0.64 (-1.02 to -0.26) | .001 |
|  | Yes | 1,010 | -1.67 (-2.92 to -0.42) | .009 |
| Interaction | Model A | 5,594 | -1.02 (-2.00 to -1.05) | .039 |
|  | Model B | 5,594 | -0.85 (-1.23 to -0.46) | <.001 |
|  | Model C | 5,121 | -1.12 (-2.12 to -0.12) | .029 |
| **Physical Symptoms 12-20 weeks** | | | | |
| Anxiety | No | 4,484 | -0.79 (-1.21 to -0.37) | <.001 |
|  | Yes | 9,80 | -1.53 (-2.97 to -0.11) | .035 |
| Interaction | Model A | 5,464 | -0.79 (-1.27 to -0.31) | .001 |
|  | Model B | 5,464 | -0.93 (-1.36 to -0.50) | <.001 |
|  | Model C | 5,001 | -0.80 (-1.29 to -0.31) | .001 |
| **Depression** | | | | |
| Anxiety | No | 4,336 | 0.55 (0.12 to 0.97) | .012 |
|  | Yes | 955 | -0.00 (-1.35 to 1.34) | 1.000 |
| Interaction | Model A | 5,291 | -0.55 (-1.63 to 0.54) | .323 |
|  | Model B | 5,291 | 0.44 (0.01 to 0.87) | .044 |
|  | Model C | 4,837 | -0.40 (-1.49 to 0.68) | .466 |
| **Anxiety** | | | | |
| Anxiety | No | 4,374 | 0.25 (-0.16 to 0.67) | .233 |
|  | Yes | 959 | -0.12 (-1.28 to 1.04) | .838 |
| Interaction | Model A | 5,333 | -0.37 (-0.19 to 0.70) | .471 |
|  | Model B | 5,333 | 0.18 (-0.22 to 0.58) | .377 |
|  | Model C | 4,883 | -0.10 (-1.13 to 0.94) | .858 |
| **Wellbeing** | | | | |
| Anxiety | No | 4,354 | -1.44 (-2.31 to -0.57) | .001 |
|  | Yes | 943 | -0.73 (-2.60 to 1.14) | .445 |
| Interaction | Model A | 5,297 | 0.77 (-1.30 to 2.72) | .488 |
|  | Model B | 5,297 | -1.30 (-2.09 to -0.51) | .001 |
|  | Model C | 4,845 | 0.17 (-1.92 to 2.26)* | .874 |

Notes: 95% CI: 95% confidence intervals. B: unstandardised coefficients. An asterisk (*) denotes an adjusted model for which the confidence intervals are consistent with a clinically significant result that our study was too small to detect.

Self-reported COVID-19 infection, coded ‘1’, is confirmed by test or suspected by participant or doctor but not tested. No COVID-19, coded ‘0’, applied to participants reporting not having had COVID-19. Long COVID composite is a composite of physical symptoms reported at 4-20 weeks after onset of COVID-19 infection, and psychological symptoms. Depression symptoms measured using the Short Mood and Feelings Questionnaire (SMFQ), anxiety symptoms measured using the Generalised Anxiety Disorder-7 questionnaire (GAD-7) and wellbeing measured using the Warwick-Edinburgh Mental Well-being Scale (WEMWS).

Linear regressions. Anxiety: unadjusted associations between confirmation of COVID-19 infection status and duration of symptoms, stratified by presence of anxiety (no vs yes). Model A: unadjusted interaction of reporting having had COVID-19 x anxiety on physical and psychological symptoms. Model B: association between reporting having had COVID-19 and physical and psychological symptoms, adjusted for anxiety. Model C: interaction of reporting having had COVID-19 x anxiety on physical and psychological symptoms, adjusted for age, sex, body mass index, ethnicity, and comorbidities at the start of the pandemic. Likelihood ratios comparing model A and model B: long COVID composite – χ^2^ 0.48, *p* =.486; physical symptoms at 4-12 weeks – χ^2^4.26, *p* =.039; physical symptoms 12-20 weeks – χ^2^ 1.74, *p* =.188; depression – χ^2^ 0.98, *p* =.323; anxiety – χ^2^ – 0.45, *p* =.471; wellbeing – χ^2^ 0.48, *p* =.488.

**Supplementary Table S11. Associations between confirmation of COVID-19 infection status on and reported physical and psychological symptoms, complete case analyses**

|  | Unadjusted | | | Adjusted | | |
| --- | --- | --- | --- | --- | --- | --- |
|  | *n* | B (95% CI) | *p*-value | *n* | B (95% CI) | *p*-value |
| Long COVID Composite | 341 | -1.51 (-3.97 to 0.95) | .229 | 341 | 0.10 (-2.26 to 2.46)* | .935 |
| **Physical Symptoms** | | | | | | |
| Physical Symptoms at 4 – 12 weeks | 523 | -1.06 (-1.93 to -0.18) | .018 | 523 | -0.65 (1.50 to 0.19) | .128 |
| Physical Symptoms at 12 – 20 weeks | 403 | -0.58 (-1.64 to 0.47) | .277 | 403 | -0.00 (-1.02 to 1.01) | .998 |
| **Psychological Symptoms** | | | | | | |
| Depression | 477 | -0.46 (-1.66 to 0.74) | .451 | 477 | -0.30 (-1.39 to 0.78) | .583 |
| Anxiety | 484 | -0.62 (-1.70 to 0.46) | .263 | 484 | -0.33 (-1.28 to 0.62) | .500 |
| Wellbeing | 482 | 1.90 (-0.15 to 3.94) | .069 | 482 | 1.43 (-0.59 to 3.44)* | .165 |

Notes: 95% CI: 95% confidence intervals. B: unstandardised coefficients. An asterisk (*) denotes an adjusted model for which the confidence intervals are consistent with a clinically significant result that our study was too small to detect.

Complete case linear regressions. Adjusted analyses are adjusted for age, sex, body mass index, ethnicity, anxiety, and comorbidities at the start of the pandemic. Confirmed COVID-19, coded ‘1’, is confirmed by any test; unconfirmed COVID-19, coded ‘0’, is suspected by participant or doctor but not tested. Long COVID composite is a composite of physical symptoms reported at 4-20 weeks after onset of COVID-19 infection, and psychological symptoms. Depression symptoms measured using the Short Mood and Feelings Questionnaire (SMFQ), anxiety symptoms measured using the Generalised Anxiety Disorder-7 questionnaire (GAD-7) and wellbeing measured using the Warwick-Edinburgh Mental Well-being Scale (WEMWS).

**Supplementary Table S12. Associations between confirmation of COVID-19 infection status and duration of COVID-19 symptoms, complete case analyses**

|  | Unadjusted | | | Adjusted | | |
| --- | --- | --- | --- | --- | --- | --- |
|  | *n* | Odds Ratio (95% CI) | p-value | *n* | Odds Ratio (95% CI) | *p*-value |
| Ongoing COVID-19 | 638 | 0.80 (0.52 to 1.22) | .291 | 638 | 0.90 (0.58 to 1.38) | .618 |
| Post-COVID-19 Syndrome | 638 | 0.69 (0.38 to 1.25) | .224 | 638 | 0.76 (0.40 to 1.42) | .388 |

Notes: 95% CI: 95% confidence intervals.

Complete case logistic regressions. Adjusted analyses are adjusted for age, sex, body mass index, ethnicity, anxiety, and comorbidities at the start of the pandemic. Confirmed COVID-19, coded ‘1’, is confirmed by any test; unconfirmed COVID-19, coded ‘0’, is suspected by participant or doctor but not tested. Ongoing COVID-19: symptoms 4 weeks or more after the onset of the original COVID-19 infection. Post-COVID-19 syndrome: symptoms 12 weeks or more after the onset of the original COVID-19 infection.

**Supplementary Table S13. Moderation by sex on the relationship between confirmation of COVID-19 infection status and physical and psychological symptoms, complete case analyses**

|  | | *n* | B (95% CI) | *p*-value |
| --- | --- | --- | --- | --- |
| **Long COVID Composite** | | | | |
| Sex | Male | 244 | -0.83 (-4.19 to 2.54) | .628 |
|  | Female | 97 | -2.40 (-5.94 to 1.15) | .182 |
| Interaction | Model A | 341 | -1.57 (-7.07 to 3.92) | .574 |
|  | Model B | 341 | -1.34 (-3.90 to 1.23) | .307 |
|  | Model C | 341 | -2.96 (-8.16 to 2.24)* | .264 |
| **Physical Symptoms 4 – 12 Weeks** | | | | |
| Sex | Male | 384 | -1.26 (-2.38 to -0.14) | .028 |
|  | Female | 139 | -0.50 (-1.97 to -0.95) | .493 |
| Interaction | Model A | 523 | 0.75 (-1.31 to 2.82) | .475 |
|  | Model B | 523 | -1.06 (-1.97 to -0.15) | .022 |
|  | Model C | 523 | 0.36 (-1.66 to 2.38)* | .726 |
| **Physical Symptoms 12-20 weeks** | | | | |
| Sex | Male | 292 | -0.42 (-1.81 to 0.96) | .550 |
|  | Female | 111 | -0.87 (-2.56 to 0.83) | .312 |
| Interaction | Model A | 403 | -0.45 (-2.86 to 1.97) | .716 |
|  | Model B | 403 | -0.55 (-1.65 to 0.55) | .323 |
|  | Model C | 403 | -0.89 (-3.23 to 1.44)* | .451 |
| **Depression** | | | | |
| Sex | Male | 335 | -0.69 (-2.14 to 0.76) | .351 |
|  | Female | 142 | 0.07 (-1.74 to 1.89) | .935 |
| Interaction | Model A | 477 | 0.76 (-1.75 to 3.27) | .551 |
|  | Model B | 477 | -0.46 (-1.61 to 0.69) | .432 |
|  | Model C | 477 | 0.90 (-1.40 to 3.20)* | .441 |
| **Anxiety** | | | | |
| Sex | Male | 339 | -0.50 (-1.89 to 0.89) | .482 |
|  | Female | 145 | -0.90 (-2.62 to 0.84) | .312 |
| Interaction | Model A | 484 | -0.39 (-2.80 to 2.02) | .750 |
|  | Model B | 484 | -0.62 (-1.72 to 0.49) | .273 |
|  | Model C | 484 | -0.33 (-2.48 to 1.82)* | .764 |
| **Wellbeing** | | | | |
| Sex | Male | 341 | 1.79 (-0.70 to 4.27) | .158 |
|  | Female | 141 | 2.13 (-1.61 to 5.88) | .262 |
| Interaction | Model A | 482 | 0.35 (-4.17 to 4.86) | .880 |
|  | Model B | 482 | 1.89 (-0.17 to 3.95) | .072 |
|  | Model C | 482 | 3.75 (-1.93 to 9.43)* | .195 |

Notes: 95% CI: 95% confidence intervals. B: unstandardised coefficients. An asterisk (*) denotes an adjusted model for which the confidence intervals are consistent with a clinically significant result that our study was too small to detect.

Confirmed COVID-19, coded ‘1’, is confirmed by any test; unconfirmed COVID-19, coded ‘0’, is suspected by participant or doctor but not tested. Long COVID composite is a composite of physical symptoms reported at 4-20 weeks after onset of COVID-19 infection, and psychological symptoms. Depression symptoms measured using the Short Mood and Feelings Questionnaire (SMFQ), anxiety symptoms measured using the Generalised Anxiety Disorder-7 questionnaire (GAD-7) and wellbeing measured using the Warwick-Edinburgh Mental Well-being Scale (WEMWS).

Complete case linear regressions. Sex: unadjusted associations between confirmation of COVID-19 infection status and duration of symptoms, stratified by sex (male vs female). Model A: unadjusted interaction of confirmation of having had COVID-19 x sex on physical and psychological symptoms. Model B: association between confirmation of having had COVID-19 and physical and psychological symptoms, adjusted for sex. Model C: interaction of confirmation of having had COVID-19 x sex on physical and psychological symptoms, adjusted for age, body mass index, ethnicity, anxiety, and comorbidities at the start of the pandemic. Likelihood ratios comparing model A and model B: long COVID composite – χ^2^ 0.32, *p* =.572; physical symptoms 4-12 weeks– χ^2^ 0.52, *p* =.473; physical symptoms 12-20 weeks – χ^2^ 0.13, *p* =.715; depression – χ^2^ 0.36, *p* =.549; anxiety – χ^2^ 0.10, *p* =.749; wellbeing – χ^2^ 0.02, *p* =.880.

**Supplementary Table S14. Moderation by sex on the relationship between confirmation of COVID-19 infection status and duration of symptoms, complete case analyses**

|  | | *n* | Odds ratio (95% CI) | *p*-value |
| --- | --- | --- | --- | --- |
| **Ongoing COVID-19** | | | | |
| Sex | Male | 451 | 0.91 (0.56 to 1.47) | .692 |
|  | Female | 187 | 0.49 (0.19 to 1.26) | .139 |
| Interaction | Model A | 638 | 0.54 (0.19 to 1.56) | .255 |
|  | Model B | 638 | 0.79 (0.52 to 1.21) | .274 |
|  | Model C | 638 | 0.50 (0.17 to 1.46) | .206 |
| **Post-COVID-19 Syndrome** | | | | |
| Sex | Male | 451 | 0.65 (0.33 to 1.26) | .202 |
|  | Female | 187 | 0.82 (0.22 to 3.09) | .775 |
| Interaction | Model A | 638 | 1.28 (0.29 to 5.62) | .748 |
|  | Model B | 638 | 0.68 (0.37 to 1.23) | .203 |
|  | Model C | 638 | 1.17 (0.26 to 5.23)* | .833 |

Notes: 95% CI: 95% confidence intervals. An asterisk (*) denotes an adjusted model for which the confidence intervals are consistent with a clinically significant result that our study was too small to detect.

Confirmed COVID-19, coded ‘1’, is confirmed by any test; unconfirmed COVID-19, coded ‘0’, is suspected by participant or doctor but not tested. Ongoing COVID-19: symptoms 4 weeks or more after the onset of the original COVID-19 infection. Post-COVID-19 syndrome: symptoms 12 weeks or more after the onset of the original COVID-19 infection.

Logistic regressions. Sex: unadjusted associations between confirmation of COVID-19 infection status and duration of symptoms, stratified by sex (male vs female). Model A: unadjusted interaction of confirmation of having had COVID-19 x sex on duration of symptoms. Model B: association between confirmation of having had COVID-19 and duration of symptoms, adjusted for sex. Model C: interaction of confirmation of having had COVID-19 x sex on duration of symptoms, adjusted for age, body mass index, ethnicity, anxiety, and comorbidities at the start of the pandemic. Likelihood ratios comparing model A and model B: ongoing COVID-19 – χ^2^ 1.37, *p* =.241; post-COVID-19 syndrome - χ^2^ 0.10, *p* =.751.

**Supplementary Table S15. Moderation by anxiety on the relationship between confirmation of COVID-19 infection status and physical and psychological symptoms, complete case analysis**

|  | | *n* | B (95% CI) | *p*-value |
| --- | --- | --- | --- | --- |
| **Long COVID Composite** | | | | |
| Anxiety | No | 274 | -0.88 (-3.16 to 1.40) | .449 |
|  | Yes | 67 | -2.19 (-12.37 to 7.99) | .668 |
| Interaction | Model A | 341 | -1.32 (-8.51 to 5.88) | .719 |
|  | Model B | 341 | -1.07 (-3.59 to 1.46) | .406 |
|  | Model C | 341 | -1.21 (-8.22 to 5.80)* | .735 |
| **Physical Symptoms 4 – 12 Weeks** | | | | |
| Anxiety | No | 421 | -0.62 (-1.47 to 0.24) | .160 |
|  | Yes | 102 | -2.53 (-5.71 to 0.66) | .118 |
| Interaction | Model A | 523 | -1.91 (-4.34 to 0.52) | .123 |
|  | Model B | 523 | -0.93 (-1.83 to -0.03) | .043 |
|  | Model C | 523 | -2.03 (-4.43 to 0.37)* | .098 |
| **Physical Symptoms 12-20 weeks** | | | | |
| Anxiety | No | 328 | -0.20 (-1.20 to 0.81) | .699 |
|  | Yes | 75 | -1.45 (-6.13 to 3.23) | .539 |
| Interaction | Model A | 403 | -1.25 (-4.62 to 2.12) | .465 |
|  | Model B | 403 | -0.35 (-1.43 to 0.74) | .532 |
|  | Model C | 403 | -1.25 (-4.58 to 2.07)* | .459 |
| **Depression** | | | | |
| Anxiety | No | 385 | -0.37 (-1.41 to 0.66) | .478 |
|  | Yes | 92 | 1.09 (-2.92 to 5.10) | .590 |
| Interaction | Model A | 477 | 1.47 (-1.60 to 4.53) | .347 |
|  | Model B | 477 | -0.16 (-1.24 to 0.93) | .776 |
|  | Model C | 477 | 1.10 (-1.91 to 4.10)* | .473 |
| **Anxiety** | | | | |
| Anxiety | No | 392 | 0.02 (-0.97 to 1.00) | .974 |
|  | Yes | 92 | -1.74 (-5.30 to 1.73) | .322 |
| Interaction | Model A | 484 | -1.75 (-4.60 to 1.09) | .227 |
|  | Model B | 484 | -0.23 (-1.23 to 0.76) | .644 |
|  | Model C | 484 | -2.19 (-5.03 to 0.64)* | .129 |
| **Wellbeing** | | | | |
| Anxiety | No | 391 | 1.01 (-1.10 to 3.13) | .347 |
|  | Yes | 91 | 3.87 (-1.57 to 9.31) | .161 |
| Interaction | Model A | 482 | 2.86 (-2.77 to 8.49) | .319 |
|  | Model B | 482 | 1.42 (-0.55 to 3.39) | .157 |
|  | Model C | 482 | 3.75 (-1.93 to 9.43)* | .195 |

Notes: 95% CI: 95% confidence intervals. B: unstandardised coefficients. An asterisk (*) denotes an adjusted model for which the confidence intervals are consistent with a clinically significant result that our study was too small to detect.

Confirmed COVID-19, coded ‘1’, is confirmed by any test; unconfirmed COVID-19, coded ‘0’, is suspected by participant or doctor but not tested. Long COVID composite is a composite of physical symptoms reported at 4-20 weeks after onset of COVID-19 infection, and psychological symptoms. Depression symptoms measured using the Short Mood and Feelings Questionnaire (SMFQ), anxiety symptoms measured using the Generalised Anxiety Disorder-7 questionnaire (GAD-7) and wellbeing measured using the Warwick-Edinburgh Mental Well-being Scale (WEMWS).

Complete case linear regressions. Anxiety: unadjusted associations between confirmation of COVID-19 infection status and duration of symptoms, stratified by presence of anxiety (no vs yes). Model A: unadjusted interaction of confirmation of having had COVID-19 x anxiety on physical and psychological symptoms. Model B: association between confirmation of having had COVID-19 and physical and psychological symptoms, adjusted for anxiety. Model C: interaction of confirmation of having had COVID-19 x anxiety on physical and psychological symptoms, adjusted for age, sex, body mass index, ethnicity, and comorbidities at the start of the pandemic. Likelihood ratios comparing model A and model B: long COVID composite – χ^2^ 0.13, *p* =.718; physical symptoms 4-12 weeks – χ^2^ 2.41, *p* =.121; physical symptoms 12-20 weeks – χ^2^ 0.54, *p* =.463; depression - χ^2^ 0.89, *p* =.345; anxiety – χ^2^ 1.47, *p* =.225; wellbeing – χ^2^ 1.00, *p* =.317.

**Supplementary Table S16. Moderation by anxiety on the relationship between confirmation of COVID-19 infection status and duration of symptoms, complete case analyses**

|  | | *n* | Odds ratio (95% CI) | *p*-value |
| --- | --- | --- | --- | --- |
| **Ongoing COVID-19** | | | | |
| Anxiety | No | 518 | 0.70 (0.44 to 1.13) | .143 |
|  | Yes | 120 | 1.54 (0.58 to 4.06) | .386 |
| Interaction | Model A | 638 | 2.19 (0.74 to 6.45) | .155 |
|  | Model B | 638 | 0.81 (0.53 to 1.23) | .322 |
|  | Model C | 638 | 0.78 (0.48 to 1.27) | .321 |
| **Post-COVID-19 Syndrome** | | | | |
| Anxiety | No | 518 | 0.52 (0.26 to 1.06) | .073 |
|  | Yes | 120 | 1.97 (0.62 to 6.28) | .253 |
| Interaction | Model A | 638 | 3.78 (0.97 to 14.74) | .056 |
|  | Model B | 638 | 0.70 (0.39 to 1.28) | .252 |
|  | Model C | 638 | 3.70 (0.92 to 14.86)* | .065 |

Notes: 95% CI: 95% confidence intervals. An asterisk (*) denotes an adjusted model for which the confidence intervals are consistent with a clinically significant result that our study was too small to detect.

Confirmed COVID-19, coded ‘1’, is confirmed by any test; unconfirmed COVID-19, coded ‘0’, is suspected by participant or doctor but not tested. Ongoing COVID-19: symptoms 4 weeks or more after the onset of the original COVID-19 infection. Post-COVID-19 syndrome: symptoms 12 weeks or more after the onset of the original COVID-19 infection.

Complete case logistic regressions. Anxiety: unadjusted associations between confirmation of COVID-19 infection status and duration of symptoms, stratified by presence of anxiety (no vs yes). Model A: unadjusted interaction of confirmation of having had COVID-19 x sex on duration of symptoms. Model B: association between confirmation of having had COVID-19 and duration of symptoms, adjusted for anxiety. Model C: interaction of confirmation of having had COVID-19 x anxiety on duration of symptoms, adjusted for age, sex, body mass index, ethnicity, and comorbidities at the start of the pandemic. Likelihood ratios comparing model A and model B: ongoing COVID-19 – χ^2^ 1.95, *p* =.162, post-COVID-19 syndrome – χ^2^ 3.43, *p* =.064

**Supplementary Table S17. Associations between self-reported COVID-19 infection status and reported physical and psychological symptoms, complete case analysis**

|  | Unadjusted | | | Adjusted | | |
| --- | --- | --- | --- | --- | --- | --- |
|  | *n* | B (95% CI) | *p*-value | *n* | B (95% CI) | *p*-value |
| Long COVID Composite | 3,271 | -0.25 (-1.32 to 0.82) | .646 | 3,271 | -0.73 (-1.79 to 0.33) | .178 |
| **Physical Symptoms** | | | | | | |
| Physical Symptoms at 4 – 12 weeks | 5,121 | -0.73 (-1.17 to -0.29) | .001 | 5,121 | -0.91 (-1.35 to -0.47) | <.001 |
| Physical Symptoms at 12 – 20 weeks | 5,001 | -0.79 (-1.26 to -0.32) | .001 | 5,001 | -0.94 (-1.41 to -0.47) | <.001 |
| **Psychological Symptoms** | | | | | | |
| Depression | 4,837 | 0.36 (-0.11 to 0.84) | .129 | 4,837 | -0.08 (-0.51 to 0.35) | .712 |
| Anxiety | 4,883 | 0.16 (-0.30 to 0.61) | .493 | 4,883 | -0.17 (-0.58 to 0.24) | .411 |
| Wellbeing | 4,845 | -1.40 (-2.24 to -0.55) | .001 | 4,845 | -0.84 (-1.65 to -0.02) | .044 |

Notes: 95% CI: 95% confidence intervals. B: unstandardised coefficients.

Complete case linear regressions. Adjusted analyses are adjusted for age, sex, body mass index, ethnicity, anxiety, and comorbidities at the start of the pandemic. Self-reported COVID-19 infection, coded ‘1’, is confirmed by test or suspected by participant or doctor but not tested. No COVID-19, coded ‘0’, applied to participants reporting not having had COVID-19. Long COVID composite is a composite of physical symptoms reported at 4-20 weeks after onset of COVID-19 infection, and psychological symptoms. Depression symptoms measured using the Short Mood and Feelings Questionnaire (SMFQ), anxiety symptoms measured using the Generalised Anxiety Disorder-7 questionnaire (GAD-7) and wellbeing measured using the Warwick-Edinburgh Mental Well-being Scale (WEMWS).

**Supplementary Table S18. Moderation by sex on the relationship between self-reported COVID-19 infection status and physical and psychological symptoms, complete case analysis**

|  | | *n* | B (95% CI) | *p*-value |
| --- | --- | --- | --- | --- |
| **Long COVID Composite** | | | | |
| Sex | Male | 2,362 | -0.07 (-1.21 to 1.08) | .910 |
|  | Female | 909 | -0.66 (-2.06 to 0.74) | .357 |
| Interaction | Model A | 3,271 | -0.59 (-2.62 to 1.43) | .567 |
|  | Model B | 3,271 | -0.23 (-1.15 to 0.68) | .615 |
|  | Model C | 3,271 | -0.12 (-2.03 to 1.79)* | .903 |
| **Physical Symptoms 4 – 12 Weeks** | | | | |
| Sex | Male | 3,659 | -0.76 (-1.27 to -0.25) | .003 |
|  | Female | 1,462 | -0.72 (-1.41 to -0.04) | .037 |
| Interaction | Model A | 5,121 | 0.04 (-0.90 to 0.97) | .940 |
|  | Model B | 5,121 | -0.75 (-1.16 to -0.34) | <.001 |
|  | Model C | 5,121 | 0.15 (-0.75 to 1.04) | .749 |
| **Physical Symptoms 12-20 weeks** | | | | |
| Sex | Male | 3,568 | -0.81 (-1.38 to -0.24) | .006 |
|  | Female | 1,434 | -0.79 (-1.54 to -0.03) | .041 |
| Interaction | Model A | 5,001 | 0.02 (-1.01 to 1.05) | .968 |
|  | Model B | 5,001 | -0.90 (-1.26 to -0.34) | <.001 |
|  | Model C | 5,001 | 0.12 (-0.87 to 1.11) | .811 |
| **Depression** | | | | |
| Sex | Male | 3,452 | 0.44 (-0.15 to 1.02) | .146 |
|  | Female | 1,385 | 0.25 (-0.53 to 1.03) | .526 |
| Interaction | Model A | 4,837 | -0.18 (-1.22 to 0.85) | .728 |
|  | Model B | 4,837 | 0.38 (-0.09 to 0.86) | .115 |
|  | Model C | 4,837 | -0.18 (-1.11 to 0.75) | .704 |
| **Anxiety** | | | | |
| Sex | Male | 3,473 | 0.08 (-0.49 to 0.65) | .786 |
|  | Female | 1,410 | 0.41 (-0.33 to 1.15) | .273 |
| Interaction | Model A | 4,883 | 0.33 (-0.66 to 1.33) | .512 |
|  | Model B | 4,883 | 0.18 (-0.28 to 0.64) | .444 |
|  | Model C | 4,883 | 0.28 (-0.60 to 1.17) | .304 |
| **Wellbeing** | | | | |
| Sex | Male | 3,457 | -1.09 (-2.12 to -0.057) | .039 |
|  | Female | 1,388 | -2.17 (-3.74 to -0.61) | .007 |
| Interaction | Model A | 4,845 | -1.08 (-2.98 to 0.82) | .263 |
|  | Model B | 4,845 | -1.41 (-2.27 to -0.54) | .001 |
|  | Model C | 4,845 | 0.17 (-1.92 to 2.26)* | .874 |

Notes: 95% CI: 95% confidence intervals. B: unstandardised coefficients. An asterisk (*) denotes an adjusted model for which the confidence intervals are consistent with a clinically significant result that our study was too small to detect.

Self-reported COVID-19 infection, coded ‘1’, is confirmed by test or suspected by participant or doctor but not tested. No COVID-19, coded ‘0’, applied to participants reporting not having had COVID-19. Long COVID composite is a composite of physical symptoms reported at 4-20 weeks after onset of COVID-19 infection, and psychological symptoms. Depression symptoms measured using the Short Mood and Feelings Questionnaire (SMFQ), anxiety symptoms measured using the Generalised Anxiety Disorder-7 questionnaire (GAD-7) and wellbeing measured using the Warwick-Edinburgh Mental Well-being Scale (WEMWS).

Complete case linear regressions. Sex: unadjusted associations between confirmation of COVID-19 infection status and duration of symptoms, stratified by sex (male vs female). Model A: unadjusted interaction of reporting having had COVID-19 x sex on physical and psychological symptoms. Model B: association between reporting having had COVID-19 and physical and psychological symptoms, adjusted for sex. Model C: interaction of reporting having had COVID-19 x sex on physical and psychological symptoms, adjusted for age, body mass index, ethnicity, anxiety, and comorbidities at the start of the pandemic. Likelihood ratios comparing model A and model B: long COVID composite – χ^2^ 0.33, *p* =.5671; physical symptoms 4-12 weeks – χ^2^ 0.01, *p* =.940; physical symptoms 12-20 weeks – χ^2^ 0.00, *p* =.968; depression – χ^2^ 0.12, *p* =.728; anxiety – χ^2^ 0.43, *p* =.512; wellbeing – χ^2^ 1.25, *p* =.263.

**Supplementary Table S19. Moderation by anxiety on the relationship between self-reported COVID-19 infection status and physical and psychological symptoms, complete case analysis**

|  | | *n* | B (95% CI) | *p*-value |
| --- | --- | --- | --- | --- |
| **Long COVID Composite** | | | | |
| Anxiety | No | 2,725 | -0.31 (-1.17 to 0.55) | .478 |
|  | Yes | 546 | -1.08 (-4.05 to 1.90) | .478 |
| Interaction | Model A | 3,271 | -0.77 (-1.00 to 1.47) | .502 |
|  | Model B | 3,271 | -0.46 (-1.34 to 0.42) | .309 |
|  | Model C | 3,271 | -0.92 (-3.11 to 1.26)* | .408 |
| **Physical Symptoms 4 – 12 Weeks** | | | | |
| Anxiety | No | 4,221 | -0.58 (-0.97 to -0.18) | .004 |
|  | Yes | 900 | -1.71 (-3.03 to -0.39) | .011 |
| Interaction | Model A | 5,121 | -1.13 (-2.15 to -0.12) | .029 |
|  | Model B | 5,121 | -0.79 (-1.20 to -0.39) | <.001 |
|  | Model C | 5,121 | -1.12 (-2.12 to -0.12)* | .029 |
| **Physical Symptoms 12-20 weeks** | | | | |
| Anxiety | No | 4,128 | -0.68 (-1.12 to -0.25) | .002 |
|  | Yes | 873 | -1.47 (-2.97 to 0.03) | .055 |
| Interaction | Model A | 5,001 | -0.79 (-1.94 to 0.37) | .180 |
|  | Model B | 5,001 | -0.83 (-1.28 to -0.38) | <.001 |
|  | Model C | 5,001 | -0.77 (-1.90 to 0.37) | .186 |
| **Depression** | | | | |
| Anxiety | No | 3,985 | 0.39 (-0.05 to 0.83) | .084 |
|  | Yes | 852 | -0.23 (-1.63 to 1.16) | .742 |
| Interaction | Model A | 4,837 | -0.62 (-1.75 to 0.50) | .277 |
|  | Model B | 4,837 | 0.27 (-0.17 to 0.71) | .231 |
|  | Model C | 4,837 | -0.18 (-1.11 to 0.75) | .704 |
| **Anxiety** | | | | |
| Anxiety | No | 4,025 | 0.11 (-0.32 to 0.54) | .602 |
|  | Yes | 858 | -0.13 (-1.35 to 1.08) | .830 |
| Interaction | Model A | 4,883 | -0.25 (-1.31 to 0.81) | .648 |
|  | Model B | 4,883 | 0.07 (-0.35 to 0.48) | .749 |
|  | Model C | 4,883 | -0.10 (-1.13 to 0.94) | .858 |
| **Wellbeing** | | | | |
| Anxiety | No | 4,004 | -1.35 (-2.25 to -0.44) | .004 |
|  | Yes | 841 | -0.93 (-2.92 to 1.06) | .358 |
| Interaction | Model A | 4,845 | 0.41 (-1.70 to 2.53) | .701 |
|  | Model B | 4,845 | -1.27 (-2.09 to -0.44) | .003 |
|  | Model C | 4,845 | 0.17 (-1.92 to 2.26)* | .874 |

Notes: 95% CI: 95% confidence intervals. B: unstandardised coefficients. An asterisk (*) denotes an adjusted model for which the confidence intervals are consistent with a clinically significant result that our study was too small to detect.

Self-reported COVID-19 infection, coded ‘1’, is confirmed by test or suspected by participant or doctor but not tested. No COVID-19, coded ‘0’, applied to participants reporting not having had COVID-19. Long COVID composite is a composite of physical symptoms reported at 4-20 weeks after onset of COVID-19 infection, and psychological symptoms. Depression symptoms measured using the Short Mood and Feelings Questionnaire (SMFQ), anxiety symptoms measured using the Generalised Anxiety Disorder-7 questionnaire (GAD-7) and wellbeing measured using the Warwick-Edinburgh Mental Well-being Scale (WEMWS).

Complete case linear regressions. Anxiety: unadjusted associations between confirmation of COVID-19 infection status and duration of symptoms, stratified by presence of anxiety (no vs yes). Model A: unadjusted interaction of reporting having had COVID-19 x anxiety on physical and psychological symptoms. Model B: association between reporting having had COVID-19 and physical and psychological symptoms, adjusted for anxiety. Model C: interaction of reporting having had COVID-19 x anxiety on physical and psychological symptoms, adjusted for age, sex, body mass index, ethnicity, and comorbidities at the start of the pandemic. Likelihood ratios comparing model A and model B: long COVID-19 composite – χ^2^ 0.45, *p* =.501; physical symptoms 4-12 weeks – χ^2^ 4.78, *p* =.029; physical symptoms 12-20 weeks – χ^2^ 1.80, *p* =.180; depression – χ^2^ 1.18, *p* =.277; anxiety – χ^2^ 0.21, *p* =.648; wellbeing – χ^2^ 0.15, p =.701.
